# Kinetic Stabilization of Blue‐Emissive Anthracenes: Phenylene Bridging Works Best

**DOI:** 10.1002/chem.202103285

**Published:** 2021-10-12

**Authors:** Marvin Nathusius, Daniel Sleeman, Junyou Pan, Frank Rominger, Jan Freudenberg, Uwe H. F. Bunz, Klaus Müllen

**Affiliations:** ^1^ Organisch-Chemisches Institut Ruprecht-Karls-Universität Heidelberg Im Neuenheimer Feld 270 69120 Heidelberg Germany; ^2^ InnovationLab Speyerer Str. 4 69115 Heidelberg Germany; ^3^ Brilliant Optoelectronic Technology Co., Ltd. Yongda Rd. 148 318020 Taizhou Zhejiang P. R. China; ^4^ Max Planck Institute for Polymer Research Ackermannweg 10 55128 Mainz Germany

**Keywords:** anthracene, organic materials, photostability, stabilization, x-ray diffraction

## Abstract

In attempts at kinetically stabilizing blue‐emissive anthracenes, a series of 9,10‐diaryl substituted derivatives were tested for their photochemical and photooxidative persistence. A major breakthrough in light fastness comes from a new bis‐*meta*‐terphenylyl substituted anthracene which is much superior to industrially relevant 9,10‐biarylated anthracenes. The key issue is the steric shielding of the anthracene core. Further, intramolecular ring closure via Yamamoto coupling furnished a doubly bridged anthracene as a “self‐encapsulated” sky‐blue emitter which is most resistant to photodegradation. The improved stabilization was corroborated by time‐resolved irradiation experiments and rationalized by X‐ray crystallography.

## Graphical abstract

**In attempts at kinetically stabilizing blue‐emissive anthracenes** a series of 9,10‐biarylated derivatives were investigated. Bis‐*meta*‐terphenylyl substitution highly increased photochemical and photooxidative stability. Ring closure to [6]cyclo‐*meta*‐hexaphenylene bridged anthracene increased protection even further resulting in an additional stabilization.

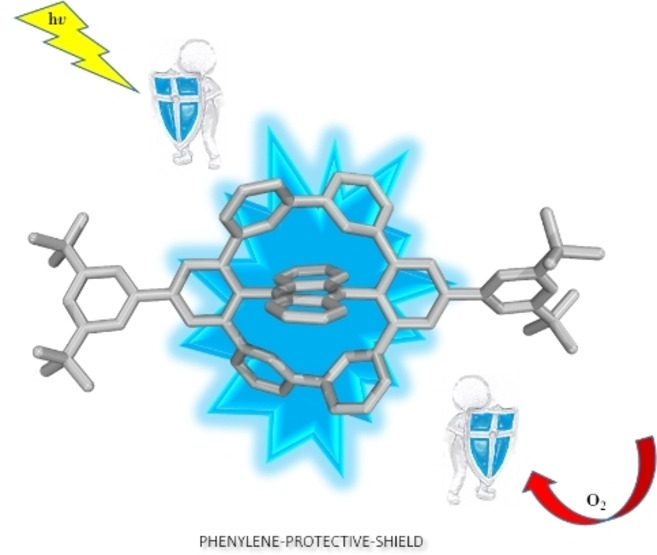

Stable blue emitters are desperately sought to improve longevity of blue OLED.^[1]^ Although anthracene‐based materials^[2]^ are of particular interest due to their high fluorescence quantum yields close to unity,^[3]^ high external device efficiencies^[4]^ as well as ease of synthesis, they still suffer from severe stability issues. Anthracene and 9,10‐diphenylanthracene are known to facilely dimerize or oxidize at the 9,10‐positions,^[5]^ hampering their application until today.[^2d^, ^4a^] There are numerous concepts towards stabilization of anthracene derivatives,^[6]^ such as the functionalization and shielding with sterically encumbering 9,10‐substituents,^[7]^ the design of rotaxanes,^[6f]^ or the assembly in capsules.[^6a^, ^6b^, ^6c^, ^6d^, ^6e^] A further development of the shielding of the reactive 9,10‐positions was presented by Kobayashi et al.,^[7a]^ who investigated doubly alkylene‐bridged diarylanthracenes which appeared to be considerably more persistent than 9,10‐diphenylanthracene. This concept was recently expanded to stabilize higher N‐acenes.^[8]^ Doubly bridged anthracenes (and N‐acenes) exhibit significantly improved resistance against (photo)oxidation and photobleaching.

The drawback of this protection concept is that it relies on electron‐rich aryl ethers which are i) photocleavable^[9]^ and, in comparison to triisopropylsilylethynylation^[10]^ as the gold standard for stabilizing the higher acenes,^[11]^ ii) promote oxidation of the aromatic core due to decreased ionization potentials.^[8]^ A second prerequisite for light fastness is the absence of strain.^[12]^ To meet these issues, we envisioned a doubly bridged anthracene with solely sp^2^‐hybridized bridging atoms:^[13]^ Our [6]cyclo‐*meta*‐hexaphenylene‐wrapped anthracene **6** ^[14]^ with shielding of the reactive 9,10‐positions (Scheme 1) is by 44–54 kcal/mol (see Supporting Information, Figure S50) less strained than its *para*‐connected analogue recently introduced by Höger et al.^[13b]^ Both the title compound **6** and its precursor **5** display significantly improved photostabilities compared to other sterically encumbered anthracene derivatives providing an ultimate solution for anthracene stability issues.

**Scheme 1:**
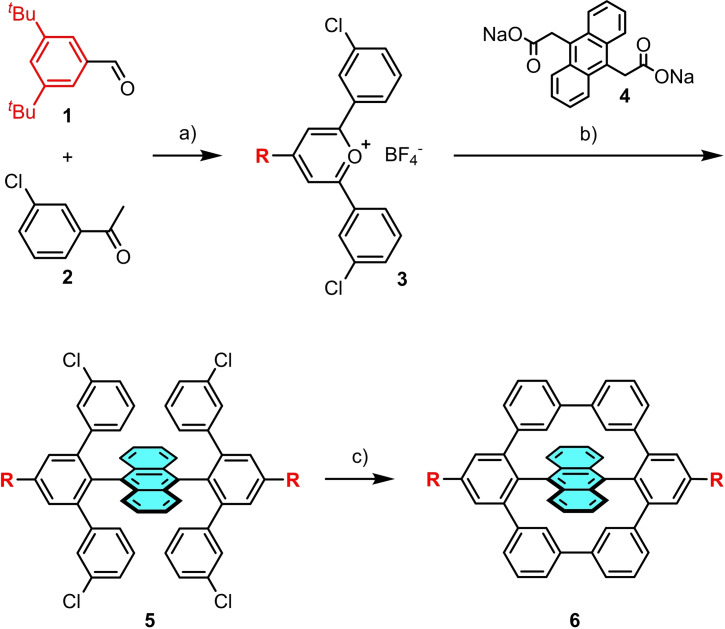
Synthetic procedure towards **6**. a) BF_3_ ⋅ OEt_2_, 80 °C, 12 h, 37 %; b) benzoic anhydride, 150 °C, 72 h, 4 %; c) Ni(COD)_2_, 2,2'‐bipyridine, THF/COD (32 : 1), 120 °C, 1 h, 50 %.

The bis‐*meta*‐terphenylyl substituted precursor **5** was synthesized via condensation reaction of pyrylium salt **3** ^[13a]^ with anthracenebisacetic acid **4** ^[15]^ (see Supporting Information, Scheme S1 for precursor synthesis).

The key reaction leading to **5** includes a carbon incorporation into the pyrylium salt with subsequent decarboxylation.^[16]^ Acetic anhydride and benzoic anhydride were used as condensation agents (see Supporting Information, Table S1 for screening reaction conditions). Using acetic anhydride in a microwave reaction for 3–6 h at 160 °C furnished only trace amounts of **5** detectable via mass spectrometry. The main product was a triphenylbenzene (see Supporting Information, Scheme S2), formed by the reaction of acetic anhydride with the pyrylium salt. With benzoic acid as a condensation agent, conventional heating at 150 °C (16 h) increased the quantity of **5** to 2.5 %.

Prolongating the reaction time to 72 h resulted in a maximum yield of 4 %–12 mg of **5** were isolated per batch. The doubly bridged target anthracene **6** was then obtained by intramolecular Yamamoto coupling in a microwave reactor. After stirring **5** for 60 min at 120 °C using Ni(COD)_2_ as catalyst and 5,5’‐bipyridyl as ligand, **6** could be isolated in a reasonable yield of 50 % as a pale yellow, crystalline solid. Shorter reaction times led to hard‐to‐separate by‐products such as the corresponding mono‐coupled intermediate (only one successful C−C ring closure) and dehalogenated species (see Supporting Information, Figure S19, identification via mass spectrometry).

Single crystals of **5** and **6** were grown by slow evaporation of a saturated THF or DCM solution under air (Figure 1). **6** crystallized with four molecules and 12 THF molecules per unit cell, while **5** contained two molecules and four DCM molecules in its unit cell (for packing see Supporting Information, Figure S51). In both crystals π‐π‐stacking was not observed, as the anthracene units were kept apart by the *meta*‐terphenyl units. The ring closure from **5** to **6** caused a distinctive twist of the phenylene units with respect to each other resulting in 4 H atoms directly facing the anthracene π‐system with a distance of only 2.27–2.32 Å. The corresponding magnetic shielding resulted in a resonance at 5.83 ppm (see Supporting Information, Figure S13+S14–16 for 2D‐spectra). In contrast to Höger's encapsulated anthracene,^[13b]^ the phenylene rings in **6** do not deviate significantly from planarity. Thus, **6** is significantly less strained,^[19]^ which is also supported by simple molecular mechanics simulations (see Supporting Information, Figure S50). Space filling models suggest that the π‐system of **6** experiences a more pronounced steric shielding than that of **5** (Figure 1c, f).

**Figure 1:**
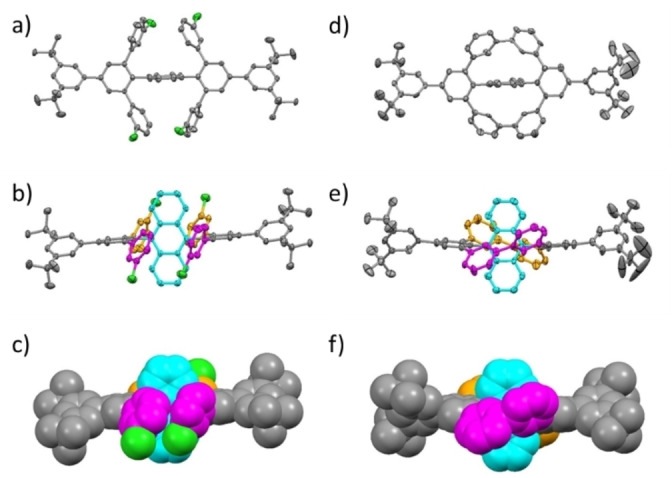
Crystal structure of **5** (left) and **6** (right).^[18]^ a, d) Side view. b, e) Top view. c, f) Top view (space filling model). Color scheme for b–f: Upwards facing arylenes: magenta; downward facing arylenes: orange; anthracenylene: cyan.

The optical properties of **5** and **6** were compared to that of Kobayashi's doubly alkynyl‐bridged reference system **11** (Table 1, Figure 2). **5** and **11** displayed quite similar absorption and emission spectra, with emission maxima at 423 nm and 407 nm, respectively. The expected red shift due to phenyl substitution of the anthracene is small due to the perpendicular orientation of anthracene core and substituents. Ring closure from **5** to **6** lead to a bathochromic shift of 41 nm (2439 cm^−1^), resulting in a sky‐blue emission for **6** compared to a purple‐blue emission of **5** (and **11**). This shift by 0.21 eV (obtained from the onsets of the absorbance) is in good agreement with theoretical calculations since the difference in frontier molecular orbital (FMO) gaps amounts to 0.22 eV. TDDFT (Time‐Dependent DFT) calculations confirm that the S_1_‐S_0_ transition of **6** predominantly corresponds to a HOMO‐LUMO transition. FMOs are mostly located on the anthracene core with small contributions from the phenylene rings located above the anthracene π‐system. (see Supporting Information, Figure S48+49). The cyclohexa‐*meta*‐phenylene alone exhibits an onset of absorption of only ca. 315 nm in chloroform.^[19]^ The red‐shifted absorption of **6** when compared to **5** is most likely a consequence of through‐space interactions since through‐bond‐conjugation between bridges and anthracene are largely hampered.

**Table 1:** Optical characterization of **5**, **6** and **11**.

Compound	λ_abs_^[a]^ [nm]	λ_em_^[a,b]^ [nm]	ϵ [L/mol*cm]	QY solution [%]	τ_f_ [ns]	HOMO/LUMO^[c]^ [eV]	λ_onset_/gap calc.^[d]^ [eV]	Stokes shift [cm^−1^]
**5**	258/403	423	8.3*10^4^	92	5.8	−5.51/−2.10	2.95/3.41	1173
**6**	261/442	464	8.7*10^4^	69	7.1	−5.17/−1.98	2.74/3.19	1072
**11**	261/396	407	7.2*10^4^	97	5.3	–	2.98/–	682

[a] All spectra were measured in DCM, wavelengths with maximum absorbance and maximum wavelength given respectively. [b] Excitation wavelength: 365 nm for **5** and **11** and 390 nm for **6**. [c] Frontier molecular orbital energies were obtained from quantum‐chemical calculations with Gaussian16 B3LYP/def2SVP//Gaussian16 B3LYP/def2TZVP.^[17]^ [d] Absorption onset in DCM.

**Figure 2:**
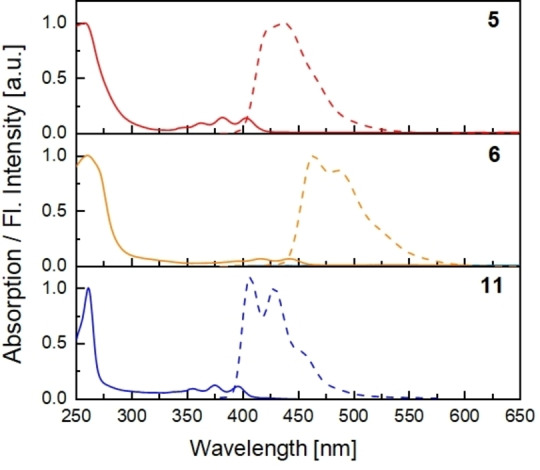
Absorption (continuous line) and emission spectra (dashed) of **5** (top), **6** (middle) and doubly alkenyl bridged anthracene **11** (bottom). All spectra were measured in DCM (10^−5^ mol/L), λ_ex_=365 nm (**5**,**11**) and λ_ex_=390 nm (**6**).

While the quantum yields (QYs) of **5** and **11** are close to unity (97 % and 92 %), it amounts to 69 % for **6**. However, as QYs were determined with the relative method using quinine sulfate as reference, an error of ±10 % must be considered.

Photodegradation of chromophores in solution depends on the excitation wavelength, the presence of oxygen as well as the solvent.^[20]^ The latter is important in terms of oxygen solubility/saturation,^[21]^ and whether reactive species (e. g. radicals) are generated from the solvent itself via photodecomposition or oxygen‐mediated degradation. Such is the case for halogenated solvents^[22]^ ‐ unselective degradation and/or halogenation may result.^[8]^ In inert solvents, two predominant primary degradation pathways have been reported for anthracene and the higher acenes: i) formation of *s*‐dimers via [4+4] cycloadditions^[23]^ or ii) *endo*‐peroxide formation.[^8^, ^24^] The latter invokes several (unselective) thermal/photochemical follow‐up reactions after homolytic O−O cleavage leading to bisepoxides, benzocyclobutendiethers, cyclic acetals, quinones,^[25]^ or Kornblum‐DeLaMare‐type rearrangements to semiquinones.^[26]^ Photostability is commonly said to improve with the shielding of the reactive acene core.[^8^, ^27^]

The photodegradation of **5**, **6** and **11** was assessed in comparison to 9,10‐diphenylanthracene **7** and industrially relevant blue anthracene emitter materials 9‐(naphthalen‐1‐yl)‐10‐(naphthalen‐2‐yl)anthracene (**8**), 2‐(10‐([1,1′‐biphenyl]‐2‐yl)anthracen‐9‐yl)naphtho[2,3‐*b*]benzofuran (**9**) and 9‐(naphthalen‐1‐yl)‐10‐(4‐(naphthalen‐2‐yl)phenyl)anthracene (**10**).^[28]^ Their dilute solutions were irradiated in quartz cuvettes (3 mL, 10^−5^ mol/L) either under ambient conditions (air atmosphere) or under argon atmosphere with degassed solvents (for details see Supporting Information, section 2.3). The photodecomposition was monitored via absorbance measurements, tracking the decrease of the most red‐shifted absorption maximum associated to the anthracene core. Figure 3 compares half‐lives (τ_1/2_; time span for absorbance to reach 50 % of its initial intensity) as a measure of kinetic stability. First, photostable cyclohexane, an ideal solvent for photobleaching experiments (see Figure 3, left), was used in combination with a Rayonet RPR‐200 photochemical reactor (16 lamps, 254 nm). Under aerated conditions, **5** and **6** were by far the most stable derivatives with half‐lives of 210 min and 368 min, respectively. The shortest τ_1/2_ of 38 min was measured for 9,10‐diphenylanthracene (**7**). This is not surprising since the phenyl substitution does not provide sufficient protection against photobleaching. Compounds **8**–**11** showed an increase in stability compared to **7** all with τ_1/2_ around 50 min as a result of higher steric demand of the substituents, such as *ortho*‐biphenylyl (**9**) or 1‐naphthalenyl (**8**, **10**). The protection of the anthracene core with the encapsulating *meta*‐terphenylyl units in **5** stabilized the chromophore by a factor of >4 compared to **7**–**11**, while the ring closure to **6** increased τ_1/2_ even further by a factor of 1.8 compared to **5**. Under argon atmosphere, no change in absolute or relative half‐lives was observed ‐ under our conditions, there is no significant degradation by oxidation (e. g. *endo*‐peroxide formation). For **5**–**11**, mass spectrometry of irradiated samples failed to show adduct formation (oxygen, dimerization), but rather unselective fragmentation. It should be noted that an irradiation wavelength of 254 nm corresponds to ∼471 kJ/mol which is higher than most bond energies involving singly bonded carbon species.^[29]^ Such conditions are thus responsible for unselective degradation.

**Figure 3:**
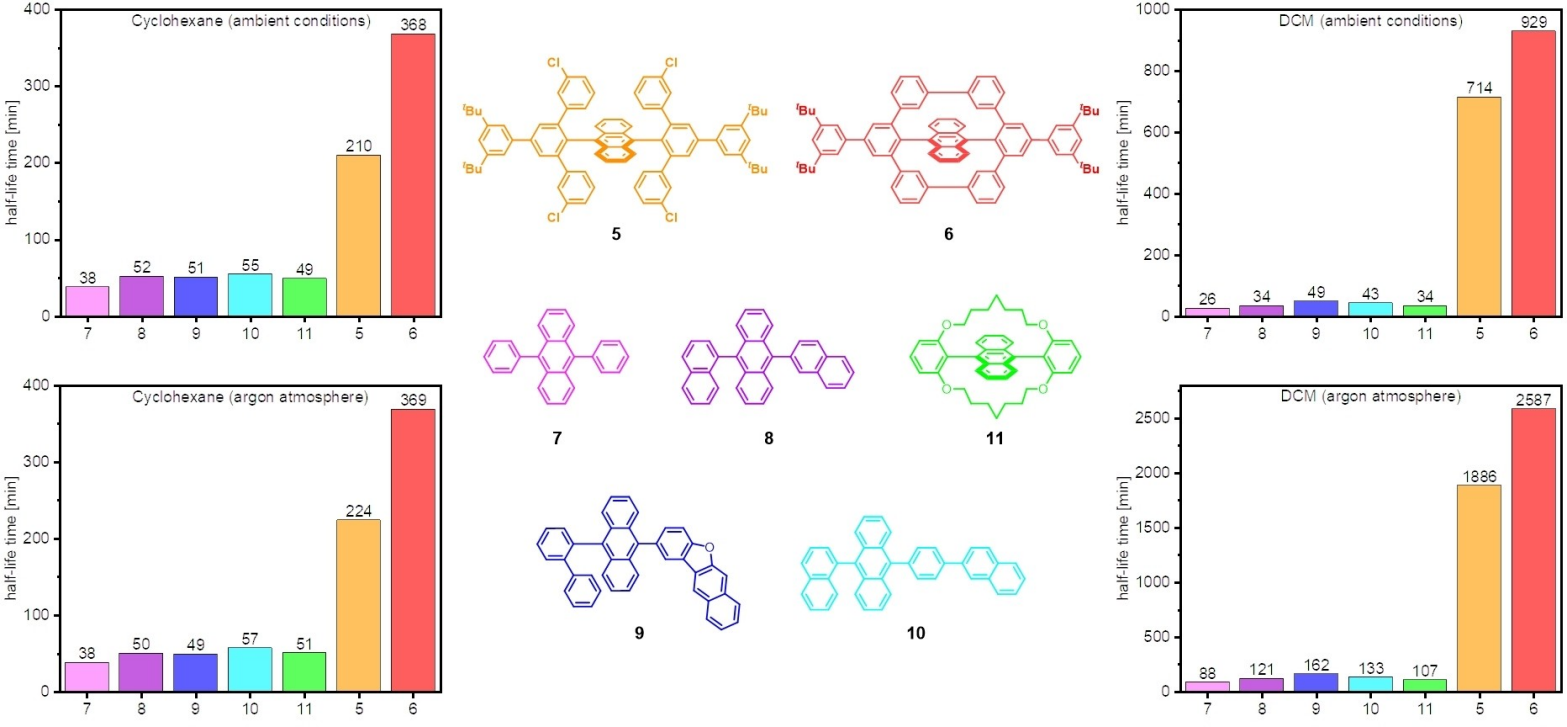
Half‐lives of **5**–**11** in cyclohexane (left, irradiation in a Rayonet RPR‐200 photochemical reactor at 254 nm) and dichloromethane (right, irradiation with a handheld UV‐lamp at 254 nm and 365 nm) under ambient conditions (top) and under argon atmosphere (bottom). All samples were irradiated in quartz cuvettes using 3 mL of solvent with a concentration of 10^−5^ mol/L (for detailed irradiation information and spectra see Supporting Information, section 2.3.1+2.3.2).

In order to provoke the bleaching process and bring about even harsher conditions, DCM was employed as a non‐innocent solvent since it undergoes radical formation under irradiation.^[22a]^ In the previous set‐up, half‐lives turned out to be too short, so that a single handheld UV‐lamp (for details, see Supporting Information, section 2.3.2 (λ_1_=254 nm; λ_2_=365 nm)) with a distance of 12 cm to the lamp was used. Again, under aerated and inert conditions, **5** and **6** were much more resistant to photobleaching than **7**–**11**. Compared to **7**–**11**, the half‐life of **5** was improved by a factor of 14–27 under ambient conditions, while ring closure to **6** further increased stability by a factor of 1.3. Under argon atmosphere, the half‐lives of **7**–**11** increased to 50 min for **7** to 162 min for **9**, while **5** and **6** showed again highly increased values of τ_1/2_ of 1886 min and 2587 min. In DCM the time‐dependent absorbance did not reach the baseline for fully decomposed samples at λ_max_ since some photoproducts with red‐shifted absorbances were formed. For **5** and **6**, unidentified adducts with +28 m/z and +48 m/z (see Supporting Information, Figures S20+S21) were observed via mass spectrometry – attempts to characterize these products via crystallography were not met with success. Compared to cyclohexane, the stability increases of all emitters under argon atmosphere leads to the conclusion that oxygen must be involved in the decomposition process under aerated conditions in DCM, although mass analysis did not reveal *endo*‐peroxide formation nor addition of radicals or halogenation for **7**–**11**.

The above solution experiments describe the intrinsic photochemical stabilities of anthracene chromophores. In view of their tremendous importance in OLEDs, two additional issues must be considered. The devices i) comprise thin solid films and ii) their lifetimes also depend upon the applied voltage:

Addressing the first issue, we have irradiated amorphous thin films of **5** and **8**–**10** on glass plates in the photoreactor (for film preparation see Supporting Information, section 2.3.3). While **8**–**10** all showed half‐lives of around 3–4 min in air, the half‐life of **5** was determined as 18 min. Please note that interpretation within this series is less valid since different film thickness may lead to slight changes of the half‐lives. However, the increased stability of **5** compared to all other samples again underlines the importance of our bridging approach. Films of **7** and **11** were omitted in our comparison as they were crystalline after solution deposition (see Supporting Information, section 2.4 for polarized light microscopy images) which resulted in scattering and increased stability compared to the amorphous samples.^[30]^

Regarding the second issue, the voltage of operation should be kept low requiring efficient charge carrier transport. Also, transport of holes and electrons should be balanced to obtain maximal internal quantum efficiency (IQE). The *meta*‐terphenylene protected anthracene compounds, though unique for their high photostability, may hamper charge carrier mobility because of their missing π‐π‐stacking (see Supporting Information, Figure S51) and cause unbalanced transport.^[31]^ In modern, multi‐layered OLEDs, host materials are responsible for charge transport and exciton formation – the combination of the fluorophores investigated herein with a host ensuring good and balanced transport should resolve this issue.

In conclusion, we present a novel *meta*‐hexaphenylene encapsulated anthracene **6** by intramolecular Yamamoto coupling of **5**. Ring closure from **5** to **6** led to skyblue emission for **6** compared to the purple‐blue emission of **5** and **11**. The target molecules **5** and **6** displayed highly increased photostability compared to anthracenes **7**–**11** due to bis‐*ortho*‐phenylation: The main stabilizing effect was achieved in **5**, which could be further increased by the internal ring closure to **6**. The differences in τ_1/2_ between **5** and **6** are comparatively small resulting from the already excellent shielding of the anthracene core by the two *meta*‐terphenylene units. Although the anthracene core seems to be significantly more protected in **6** than in **5** as visualized by the single crystal structures, one has to keep in mind that different conformations of the 3‐chlorophenyl substituents are possible in solution due to rotation around the single bonds providing improved shielding as compared to the solid‐state conformation. Our concept of *meta*‐terphenylation or cyclo‐*meta*‐phenylene‐encapsulation should not only be transferable to other chromophores impeding photobleaching, but they should also allow the stabilization of otherwise fairly sensitive systems such as the higher acenes or cyclacenes. This model study is targeting the limits of molecular design in attempts at kinetically stabilizing the important anthracene chromophore and related systems. The practical use is certainly hampered by the low‐yielding synthesis. On the other hand, there are many alternative options for cross‐coupling reactions providing mono‐ or bis‐*meta*‐terphenyl substituted chromophores.

## Conflict of interest

The authors declare no conflict of interest.

## Supporting information

As a service to our authors and readers, this journal provides supporting information supplied by the authors. Such materials are peer reviewed and may be re‐organized for online delivery, but are not copy‐edited or typeset. Technical support issues arising from supporting information (other than missing files) should be addressed to the authors.

Supporting Information
