# Supplementary material for: Kinetic Stabilization of Blue‐Emissive Anthracenes: Phenylene Bridging Works Best
Source: Chemistry. 2021 Oct 12;27(67):16606–10. doi: 10.1002/chem.202103285 (PMC9293334; doi:10.1002/chem.202103285)
Supplement: Supplementary file 1 — Supporting Information [file CHEM-27-16606-s001.pdf]

Chemistry–A European Journal

Supporting Information

Kinetic Stabilization of Blue-Emissive Anthracenes: Phenylene Bridging Works Best

Marvin Nathusius, Daniel Sleeman, Junyou Pan, Frank Rominger, Jan Freudenberg,* Uwe H. F. Bunz,* and Klaus Müllen*

Table of Contents

Table of Contents 1
1 Experimental Procedure 2
1.1 Materials and Methodes 2
1.2 Synthetic Procedure 4
2. Results and Discussion 12
2.1 NMR Spectra 12
2.2 Mass Analysis 20
2.3 Stability Measurements 23
2.3.1 Stability Measurements in Cyclohexane 23
2.3.2 Stability Measurements in Dichloromethane 27
2.3.3 Stability Measurements of thin films 31
2.4 Polarized Light Micrographs 32
2.5 Calculations 34
2.6 Crystallographic data 36
References 39

1 Experimental Procedure

1.1 Materials and Methodes

Reagents and Solvents for Synthesis

All reagents/solvents for synthesis were obtained from commercial suppliers and used without further purification. Deuterated solvents for NMR analysis were purchased from Sigma-Aldrich Laborchemikalien GmbH. Absolute solvents were used directly from a solvent purification system (MB SPS-800) or commercial anhydrous solvents taken from a glovebox.

Column Chromatography

Flash column chromatography was carried out using silica gel (grain size 0.04 - 0.063 mm) by Sigma-Aldrich. For the mobile phases the solvents named in the synthetic procedure were used. For thin layer chromatography Polygram Sil g/UV 254 plates from Macherey Nagel were used and examined under UV-light (254 nm or 365 nm) or stained with the help of coloring reagents.

Nuclear Magnetic Resonance Spectroscopy

All NMR spectra were recorded in deuterated solvents (DMSO-d_6, $CDCl_3$, THF-d_8) at room temperature, either on a Bruker Avance III (300 MHz), Bruker Avance III (400 MHz), Bruker Avance III (600 MHz) or Bruker Avance III (700 MHz). ^{13}C NMR spectra were measured with proton decoupling if not stated otherwise. Chemical shifts δ are reported in part per million (ppm) and coupling constants J in Hz. All spectra were referenced to solvent signal.[S1] For the multiplicities, the following abbreviations are used: s = singlet, d = doublet, t = triplet, m = multiplet. The spectra were processed and integrated using ACD/Spectrus processor.

Mass Spectrometry

High-resolution mass spectra (HRMS) were obtained by matrix-assisted laser desorption ionization (MALDI) using DCTB as matrix and a Bruker AutoFlex Speed time-of-flight spectrometer.

IR Spectra

IR spectra were recorded on a JASCO FT/IR-4100 using the neat compounds at room temperature. The data was processed using JASCO Spectra Manager™ II and all signals are reported in wavenumbers [cm^{-1}].

Microwave

All reactions were done in an Anton Paar Monowave 400 microwave reactor.

X-ray Single-Crystal Structure Analysis

X-ray single-crystal structure analyses were measured on a STOE Stadivari CCD area detector diffractometer. Diffraction intensities were corrected for Lorentz and polarization effects. An empirical scaling and absorption correction was applied using X-Area LANA 1.70.0.0 based on the Laue symmetry of reciprocal space, structures were solved with SHELXT-2014 [S2] and refined against F2 with a Full-matrix least-squares algorithm using the SHELXL-2018/3 (Sheldrick, 2018) software.[S3]

Quantum Yields

Quantum yields were measured using quinine hemisulfate salt monohydrate in a 1 N H_2SO_4 solution[S4] with a refractive index of 1.346 and a reference quantum yield of 54%.[S4] All samples were measured in DCM as solvent.

UV-Vis and Fluorescence Spectra

All UV-Vis spectra were recorded on a JASCO UV-Vis 660. Fluorescence spectra were recorded on a Jasco FP-6500 in the stated solvents.

Computational Studies

Computational studies were carried out using DFT calculations on Turbomole 6.3.1 and Gaussian16.[S5] Geometry optimizations were performed using the B3LYP functional and def2-TZVP basis set. At this geometry, the absolute energy and FMO energies were assigned by a single-point approach at the B3LYP/6-311++G** level of theory.

Melting Points

Melting points were determined in open glass capillaries with a Melting Point Apparatus MEL-TEMP (Electrothermal, Rochford, UK).

Stability Measurements (Solution)

All UV-vis stability studies were performed irradiating dilute solutions (10^{-5}mol/L in 3.00 mL cyclohexane or dichloromethane) of the sample in a quartz cuvette at room temperature.

- Irradiation of all samples in cyclohexane was performed in a Rayonet RPR-200 photochemical reactor (16 lamps, 254 nm). All samples were placed in the middle of the photoreactor with a distance of 10 cm to the lamps.

- Irradiation of all samples in dichloromethane was performed using a handheld UV-lamp from HEROLAB GmbH LABORGERÄTE, Wiesloch (type: NU-15, λ_1= 254 nm (15W) and λ_2= 365 nm (15W)). All samples were irradiated with 254 nm and 365 nm simultaneously with a distance of 12 cm to the lamp.

For all stability measurements cyclohexane and dichloromethane (HPLC grades) were used. Measurements under ambient conditions were performed under air atmosphere while measurements under argon atmosphere were performed in flame dried inert gas quartz cuvettes. For all measurements under argon atmosphere, the solvents were degassed for 2 h by bubbling an argon stream **through** the solution (in a flame dried schlenk flask) before the measurement.

For all stability measurements the percentage progression was determined at the most red shifted acene signal (belonging to anthracene; $\lambda_{abs,max}$). The measurement at 0 min was set to be 100%.

Film Preparation

Thin films were processed using chloroform as solvent (concentration: 10 mg in 3 mL). Menzel glasses were used as slides (1x2 cm) and films were obtained by spin coating of the solutions at 1000 rpm for 40 s.

1.2 Synthetic Procedure

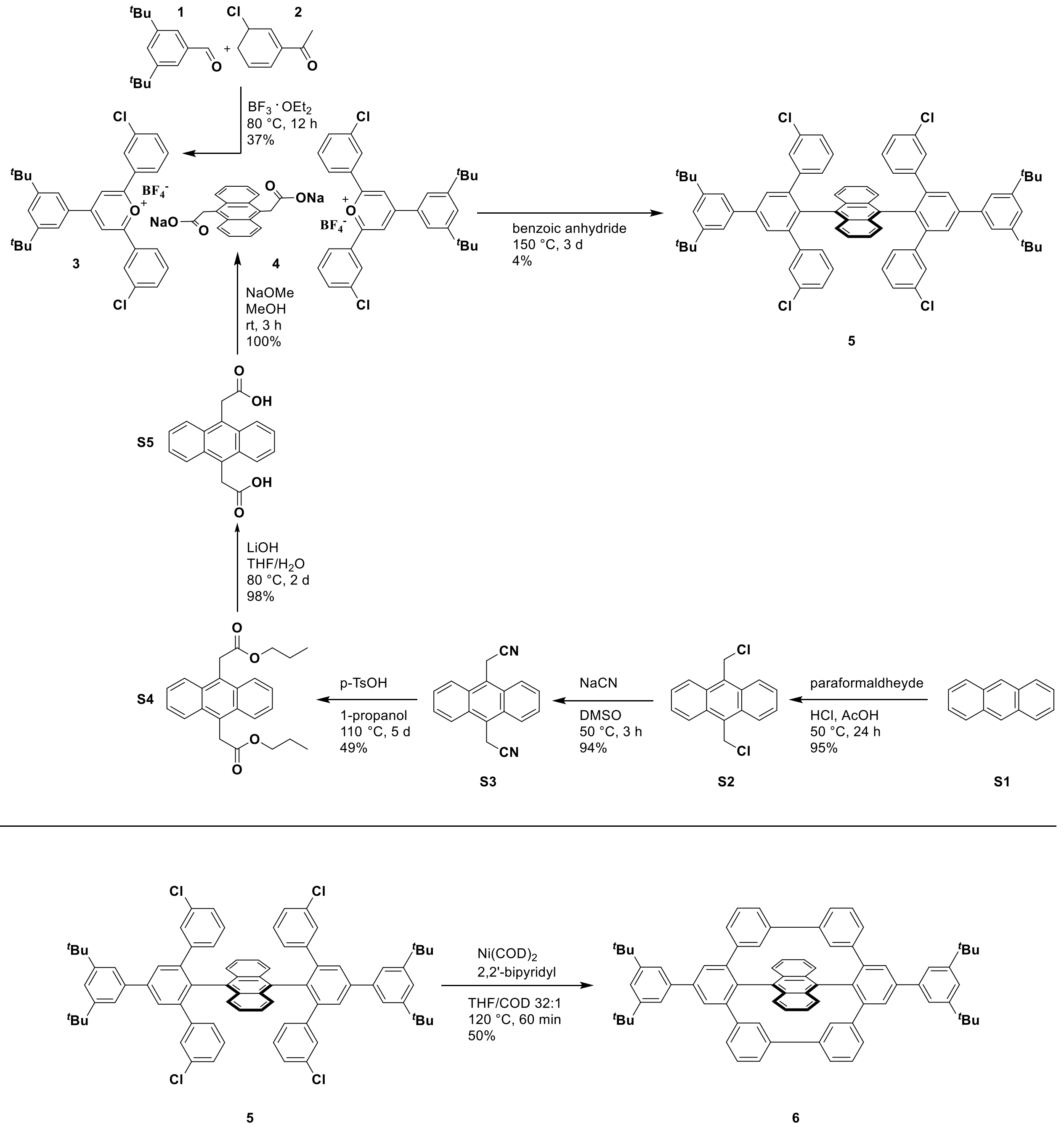

Scheme S1: Detailed synthetic procedure for the synthesis of starting materials **3**, **4** and **5**, **6**.

The Kobayashi system **11** [S6] and industrially relevant anthracenes **8-10** [S7] were synthesized according to literature procedures.

9,10-Bis(chloromethyl)anthracene (S2)

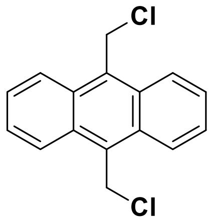

S2

Anthracene **S1** (20.0 g, 112 mmol, 1.00 eq.) and paraformaldehyde (16.8 g, 561 mmol, 5.00 eq.) were dissolved in concentrated HCl (57.0 mL, 673 mmol, 6.00 eq.) and 350 mL of acetic acid. The reaction mixture was stirred at 50 °C for 24 h, allowed to cool to room temperature and the precipitate was collected by filtration. The crude product was washed several times with H_2O to obtain a yellowish solid (29.4 g, 107 mmol, 95%). The product **S2** was used without further purification for the synthesis of **S3**.[S8]

2,2'-(Anthracene-9,10-diyl)diacetonitrile (S3)

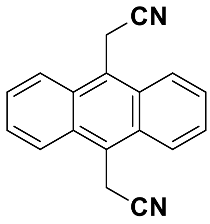

S3

9,10-Bis(chloromethyl)anthracene **S2** (12.0 g, 43.6 mmol, 1.00 eq.) and NaCN (10.7 g, 218 mmol, 5.00 eq.) were dissolved in 130 mL DMSO and stirred at 50 °C for 3 h. The reaction mixture was allowed to cool to room temperature and 1.3 L of H_2O were added to the reaction mixture. The solution was cooled in the refrigerator for 3 h before the precipitate was filtered off and washed with H_2O several times. The product **S3** was obtained as a yellow solid (10.5 g, 41.0 mmol, 94%).

^{1}H NMR (600 MHz, DMSO-d_6, 295 K): δ [ppm] = 8.52 (q, J = 3.30 Hz, 4H), 7.76 (q, J = 3.19 Hz, 4H), 5.05 (s, 4H).

^{13}C{^{1}H} NMR (151 MHz, DMSO-d_6, 295 K): δ [ppm] = 129.3, 127.0, 124.9, 124.6, 119.1, 16.0 ppm.

All analytical data is in good agreement with the literature.[S8]

Dipropyl 2,2'-(anthracene-9,10-diyl)diacetate (S4)

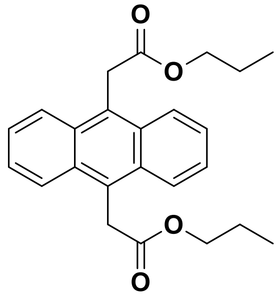

S4

2,2'-(Anthracene-9,10-diyl)diacetonitrile **S3** (11.0 g, 42.9 mmol, 1.00 eq.) was dissolved in 100 mL *n*-propanol and *p*-TsOH (73.9 g, 429 mmol, 10.0 eq.) was added slowly in portions at room temperature. The reaction mixture was stirred at 110 °C for 5 d and then allowed to cool to room temperature. The reaction was quenched with 100 mL saturated $NaHCO_3$ solution, extracted three times with DCM (3 x 150 mL), dried over Na_2SO_4 and the solvent was removed under reduced pressure. The crude product was dissolved in a small amount of DCM and poured into *n*-hexane (500 mL). The solution was cooled in the refrigerator for 3 h before the precipitate was filtered off and washed several times with *n*-hexane, the procedure was repeated twice. The product **S4** was obtained as a yellow solid (8.01 g, 21.1 mmol, 49%).

^{1}H NMR (300 MHz, $CDCl_3$, 295 K): δ [ppm] = 8.37 (q, *J* = 3.30 Hz, 4H), 7.58 (q, *J* = 3.28 Hz, 4H), 4.67 (s, 4H), 4.05 (t, *J* = 6.65 Hz, 4H), 1.58 (q, *J* = 7.03 Hz, 4H), 0.82 (t, *J* = 7.73 Hz, 6H).

^{13}C{^{1}H} NMR (75 MHz, $CDCl_3$, 295 K): δ [ppm] = 171.4, 130.4, 126.8, 125.7, 125.2, 66.6, 34.4, 21.9, 10.2.

All analytical data is in good agreement with the literature.[S8]

2,2'-(Anthracene-9,10-diyl)diacetic acid (S5)

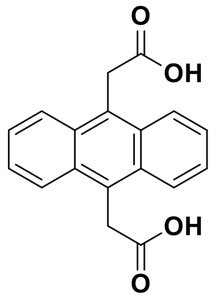

S5

Dipropyl 2,2'-(anthracene-9,10-diyl)diacetate **S4** (1.80 g, 4.68 mmol, 1.00 eq.) was dissolved in 52 mL THF/H_2O (97/3, *v*/*v*) and LiOH·H_2O (2.28 g, 95.1 mmol, 20.0 eq.) was added. The reaction mixture was stirred at 80 °C for 2 d and allowed to cool to room temperature. THF was removed under reduced pressure and the reaction mixture was poured into ice-cold H_2O. The precipitate was filtered off and the filtrate acidified with concentrated HCl. The formed precipitate was collected by filtration and washed several times with H_2O and small amounts of *n*-hexane to obtain **S5** as a pale yellow solid (1.32 g, 4.59 mmol, 98%).

^{1}H NMR (400 MHz, DMSO-d_6, 295 K): δ [ppm] = 12.49 (bs, 2H), 8.35 (q, *J* = 3.28 Hz, 4H), 7.59 (q, *J* = 3.24 Hz, 4H), 4.65 (s, 4H).

^{13}C{^{1}H} NMR (100 MHz, DMSO-d_6, 295 K): δ [ppm] = 172.6, 129.9, 127.5, 125.6, 125.2, 33.7.

All analytical data is in good agreement with the literature.[S8]

Sodium 2,2'-(anthracene-9,10-diyl)diacetate (4)

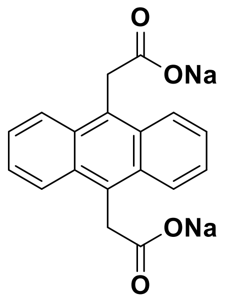

4

In an oven dried schlenk flask, 2,2'-(anthracene-9,10-diyl)diacetic acid **S5** (500 mg, 1.70 mmol, 1.00 eq.) was dissolved in 30 mL MeOH under an argon atmosphere. NaOMe in MeOH (5.4 M, 720 µL, 3.91 mmol, 2.30 eq.) was added dropwise at room temperature and the reaction mixture was stirred for an additional 3 h. The solvent was removed under reduced pressure to obtain **4** as a pale brown solid (quant.). The product was used without further purification.

2,6-Bis(3-chlorophenyl)-4-(3,5-di-tert-butylphenyl)pyrylium tetrafluoroborate (3)

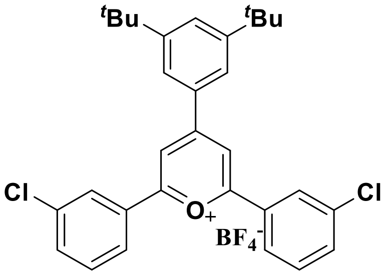

3

In an oven dried schlenk flask, $BF_3{\cdot}OEt_2$ (48% solution in Et_2O, 10.3 mL, 40.1 mmol, 3.50 eq.) was added dropwise under an argon atmosphere to a mixture of **1** (2.50 g, 11.5 mmol, 1.00 eq.) and **2** (3.27 mL, 25.2 mmol, 2.20 eq.). The reaction mixture was stirred at 80 °C for 48 h and allowed to cool to room temperature. The slurry was poured into cold Et_2O and cooled in the refrigerator for additional 2 h. The formed precipitate was collected by filtration and washed carefully several times with cold Et_2O to obtain **3** as a yellow powder (2.44 g, 4.23 mmol, 37%).

^{1}H NMR (400 MHz, DMSO-d_6, 295 K): δ [ppm] = 9.14 (s, 2H), 8.62 (t, *J* = 1.88 Hz, 2H), 8.51-8.49 (m, 2H), 8.19 (d, *J* = 1.57 Hz, 2H), 7.97-7.94(m, 2H), 7.89 (t, *J* = 1.54 Hz, 1H), 7.85 (t, *J* = 8.05 Hz, 2H), 1.45 (s, 18H).

^{13}C{^{1}H} NMR (100 MHz, DMSO-d_6, 295 K): δ [ppm] = 168.7, 167.2, 152.5, 134.5, 134.4, 132.7, 131.7, 131.1, 129.2, 128.5, 127.7, 124.5, 117.3, 35.1, 31.1.

All analytical data is in good agreement with the literature.[S9]

9,10-Bis(3,3''-dichloro-5'-(3,5-di-tert-butylphenyl)-[1,1':3',1''-terphenyl]-2'-yl)anthracene (5)

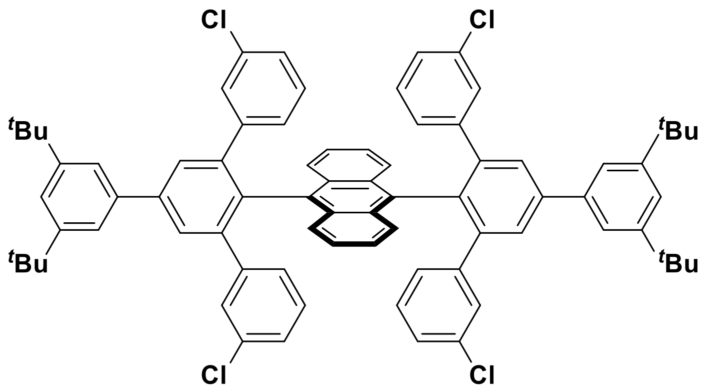

5

In an oven dried schlenk flask, a mixture of **3** (300 mg, 520 µmol, 1.00 eq.), **4** (87.9 mg, 260 µmol, 0.50 eq.) and benzoic anhydride (793 mg, 3.51 mmol, 6.75 eq.) was stirred while drying under high vacuum at room temperature for 1 h. The resulting dried powder was stirred in a pre-heated oil path at 150 °C for 72 h and allowed to cool to room temperature. The crude reaction mixture was purified by column chromatography (SiO_2, DCM) followed by a second column chromatography (SiO_2, PE/DCM 6:1). The product was obtained as a colorless solid (12.0 mg, 11.8 µmol, 4%).

R_f = 0.17 (PE/DCM 7:1)

Melting point: >300 °C

^{1}H NMR (600 MHz, $CDCl_3$, 295 K): δ [ppm] = 7.60-7.58 (m, 8H), 7.53 (d, *J* = 1.78 Hz, 4H), 7.50 (t, *J* = 1.85 Hz, 2H), 7.32-7.30 (m, 4H), 7.09 (t, *J* = 1.93 Hz, 4H), 6.89-6.87 (m, 4H), 6.45 (t, *J* = 7.85 Hz, 4H), 6.42-6.40 (m, 4H), 1.41 (s, 36H).

^{13}C{^{1}H} NMR (151 MHz, $CDCl_3$, 295 K): δ [ppm] = 151.6, 143.3, 143.2, 142.3, 139.6, 133.8, 133.7, 133.0, 130.3, 129.0, 128.7, 128.4, 127.0, 126.8, 126.1, 125.4, 122.2, 121.9, 35.3, 31.8.

IR (ATR): $\tilde{\nu}$ [cm^{-1}] = 3060, 2959, 2923, 2857, 1593, 1564, 1478, 1363, 1260, 1247, 1095, 1078, 1051, 1027.

HRMS ($MALDI^+$, DCTB): m/z: $[M+H]^+$: calcd. for $[C_{78}H_{71}Cl_4]^+$: 1149.4275; found 1149.4309; correct isotope distribution.

Crystal data

Plank-shaped, colorless single crystals were obtained by slow evaporation of a concentrated CH_2Cl_2 solution of **5**.

Colourless crystal (plank), dimensions 0.110 x 0.044 x 0.015 mm^3, crystal system triclinic, space group P $\bar{1}$, Z=1, *a*=10.2901(5) Å, *b*=12.8491(7) Å, *c*=13.6417(7) Å, α=78.112(4) °, β=83.437(4) °, γ=82.864(4) °, V=1744.01(16) $Å^3$, ρ=1.256 g/cm^3, T=200(2) K, θ_{max}= 69.182 °, 17025 reflections measured, 6260 unique (R_{int}=0.0444), 3855 observed (I > 2σ(I)), μ=3.28 mm^{-1}, T_{min}=0.76, T_{max}=10.42, 431 parameters refined, hydrogen atoms were treated using appropriate riding models, goodness of fit 1.04 for observed reflections, final residual values R1(F)=0.058, wR(F^2)=0.151 for observed reflections, residual electron density -0.51 to 0.43 e$Å^{-3}$.

Reaction conditions

Table S1: Screening of reaction conditions for condensation reaction to **5**.

entry	condensation agent	reaction conditions	result
**1**	acetic anhydride	160 °C/ 1.5 h / microwave	**S6**[b]
**2**	acetic anhydride	160 °C/ 3 h/ microwave	**S6**[c] + traces of **5**[d]
**3**	acetic anhydride	160 °C/ 6 h/ microwave[a]	**S6**[c] + traces of **5**[d]
**4**	acetic anhydride	160 °C/72 h/ oil bath[a]	**S6**[c]
**5**	benzoic anhydride	160 °C/ 3 h / microwave	--
**6**	benzoic anhydride	160 °C/ 3 h/ microwave[a]	traces of **5**[d]
**7**	benzoic anhydride	150 °C/ 3 h/ oil bath[a]	--
**8**	benzoic anhydride	150 °C/ 16 h/ oil bath[a]	2.5%
**9**	**benzoic anhydride**	**150 °C/ 72 h/ oil bath**[a]	**4%**
**10**	benzoic anhydride	200 °C/ 4 h/ microwave[a]	traces of **5**[d]

[a] Pre-dried under high vacuum for 1 h; [b] identified by ^{1}H NMR analysis (300 MHz); [c] identified by TLC (SiO_2) using PE/DCM 6:1 as eluent; [d] identified by mass analysis (MALDI$^+$, DCTB).

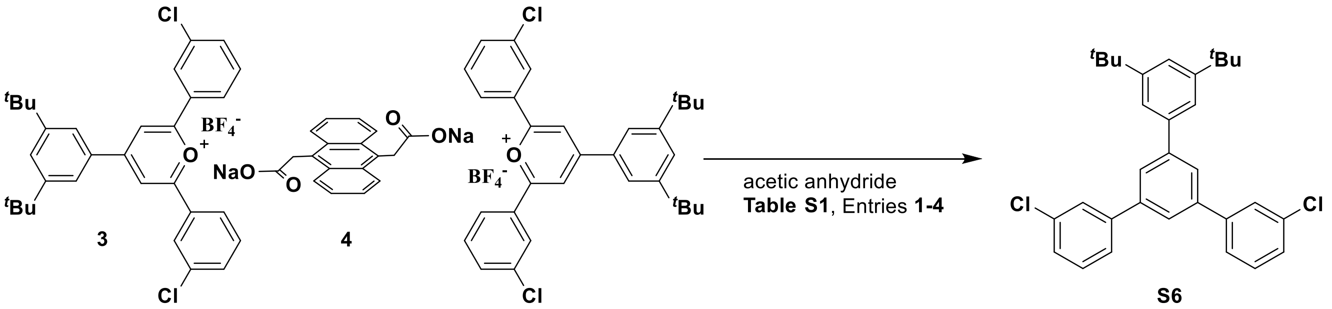

Scheme S2: Side reaction of the condensation reaction to **5** using acetic anhydride as condensation agent.

Tetra-*tert*-butyl-(cyclohexa-m-phenylen) bridged anthracene (6)

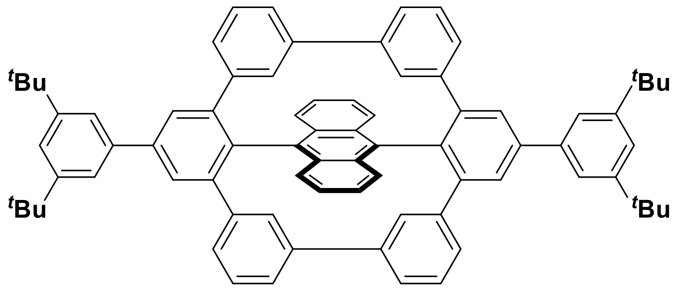

6

In a flame dried microwave vessel in a glove box, **5** (8.00 mg, 6.96 µmol, 1.00 eq.) was dissolved in 8 mL THF/COD (32:1; *v*/*v*) before 2,2-bipyridyl (10.9 mg, 69.6 µmol, 10.0 eq.) and $Ni(COD)_2$ (8.27 mg, 69.6 µmol, 10.0 eq.) were added. The reaction mixture was stirred in the microwave at 120 °C for 60 min and allowed to cool to room temperature. The crude slurry was extracted three times with DCM (3 x 15 mL), the organic layers were combined and the solvents were removed under reduced pressure. The crude product was purified by column chromatography (SiO_2, PE/DCM 6:1) to obtain product **6** as a pale yellow solid (3.50 mg, 6.96 µmol, 50%).

R_f = 0.21 (PE/DCM 6:1)

Melting point: >300 °C

^{1}H NMR (600 MHz, THF-d_8, 295 K): δ [ppm] = 8.20 (s, 4H), 7.78 (d, *J* = 1.82 Hz, 4H), 7.60 (t, *J* = 1.62 Hz, 2H), 7.48-7.47 (m, 4H), 7.10-7.08 (m, 4H), 6.94 (t, *J* = 7.65 Hz, 4H), 6.90-6.88 (m, 4H), 6.64 (d, *J* = 7.54 Hz, 4H), 5.83 (t, *J* = 1.70 Hz, 4H), 1.49 (s, 36H).

^{13}C{^{1}H} NMR (151 MHz, THF-d_8, 295 K): δ [ppm] = 151.9, 145.0, 143.5, 142.0, 141.0, 139.2, 138.6, 138.0, 135.0, 130.9, 128.9, 126.9, 126.6, 126.4, 126.3, 125.2, 122.7, 122.2, 35.6, 31.8.

IR (ATR): $\tilde{\nu}$ [cm^{-1}] = 2952, 2921, 2852, 1588, 1579, 1567, 1465, 1463, 1364, 1262, 1083, 1025.

HRMS (MALDI$^+$, DCTB): m/z: $[M]^+$: calcd. for $[C_{78}H_{70}]^+$: 1006.5473; found 1006.5472; correct isotope distribution.

Crystal data

Brick-shaped, pale yellow single crystals were obtained by slow evaporation of a concentrated THF solution of **6**.

Pale yellow crystal (brick), dimensions 0.057 x 0.027 x 0.020 mm^3, crystal system orthorhombic, space group $Pna2_1$, Z=4, *a*=19.7269(11) Å, *b*=25.1834(13) Å, *c*=14.2934(8) Å, α=90 °, β=90 °, γ=90 °, V=7100.8(7) Å^3, ρ=1.145 g/cm^3, T=200(2) K, θ_{max}= 47.235 °, 15722 reflections measured, 5980 unique (R_{int}=0.0444), 2458 observed (I > 2σ(I)), μ=0.51 mm^{-1}, T_{min}=0.70, T_{max}=1.40, 850 parameters refined, hydrogen atoms were treated using appropriate riding models, goodness of fit 0.82 for observed reflections, final residual values R1(F)=0.055, wR(F^2)=0.058 for observed reflections, residual electron density -0.16 to 0.15 eÅ^{-3}.

2. Results and Discussion

2.1 NMR Spectra

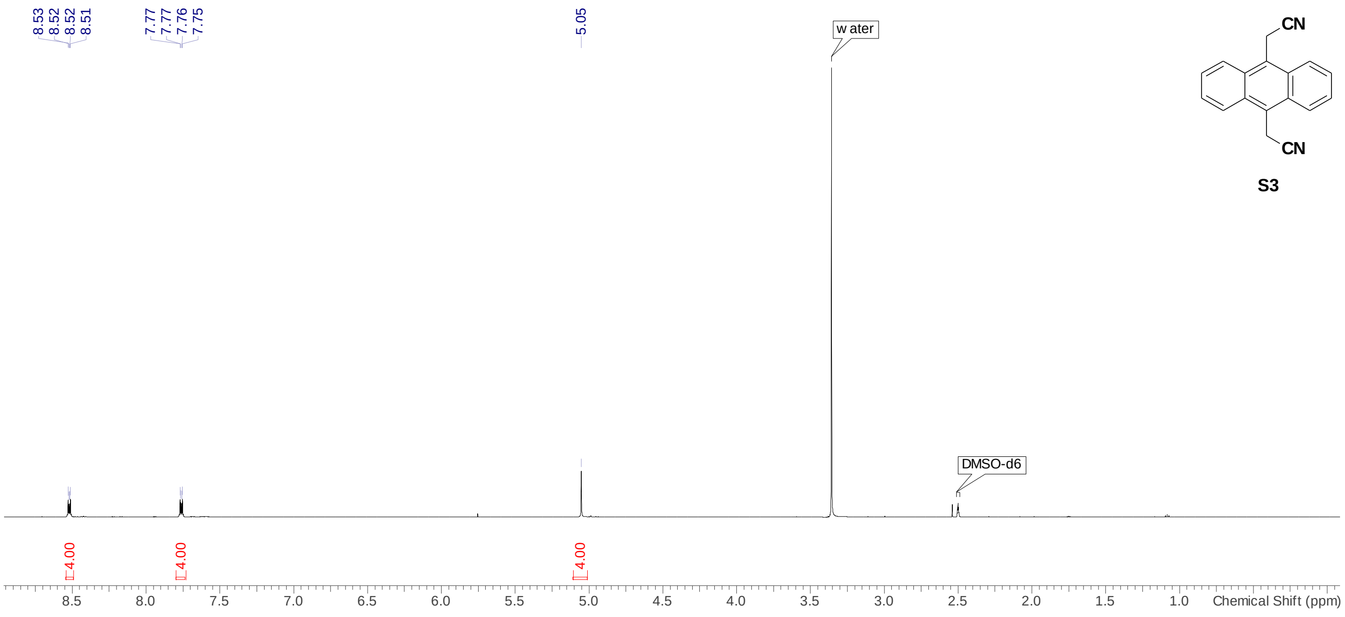

Figure S1: ^{1}H NMR spectrum (600 MHz, 295 K) of **S3** in DMSO-d_6.

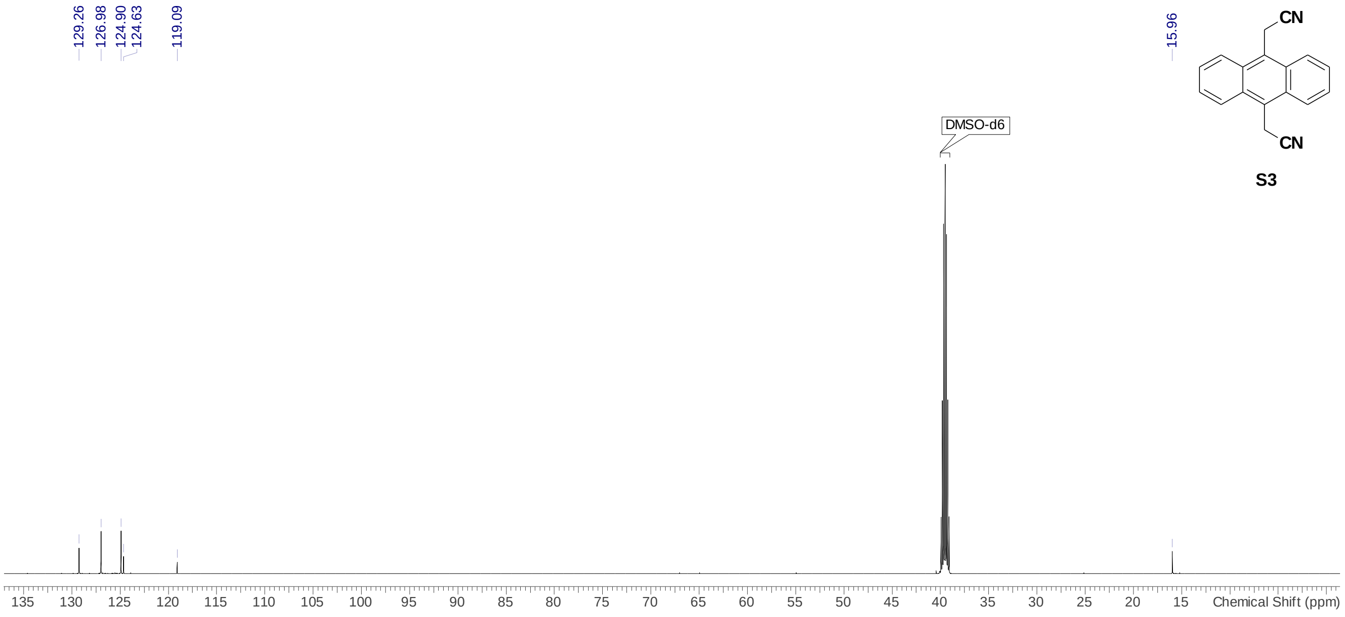

Figure S2: $^{13}C\{^{1}H\}$ NMR spectrum (151 MHz, 295 K) of **S3** in DMSO-d_6.

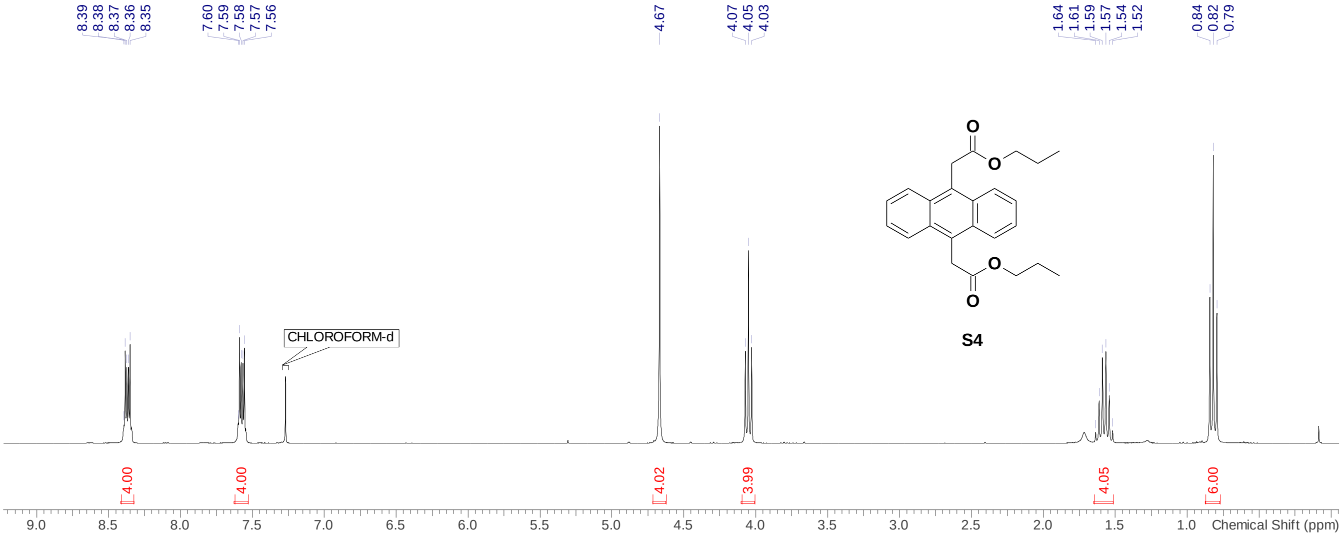

Figure S3: ^{1}H NMR spectrum (300 MHz, 295 K) of **S4** in $CDCl_3$.

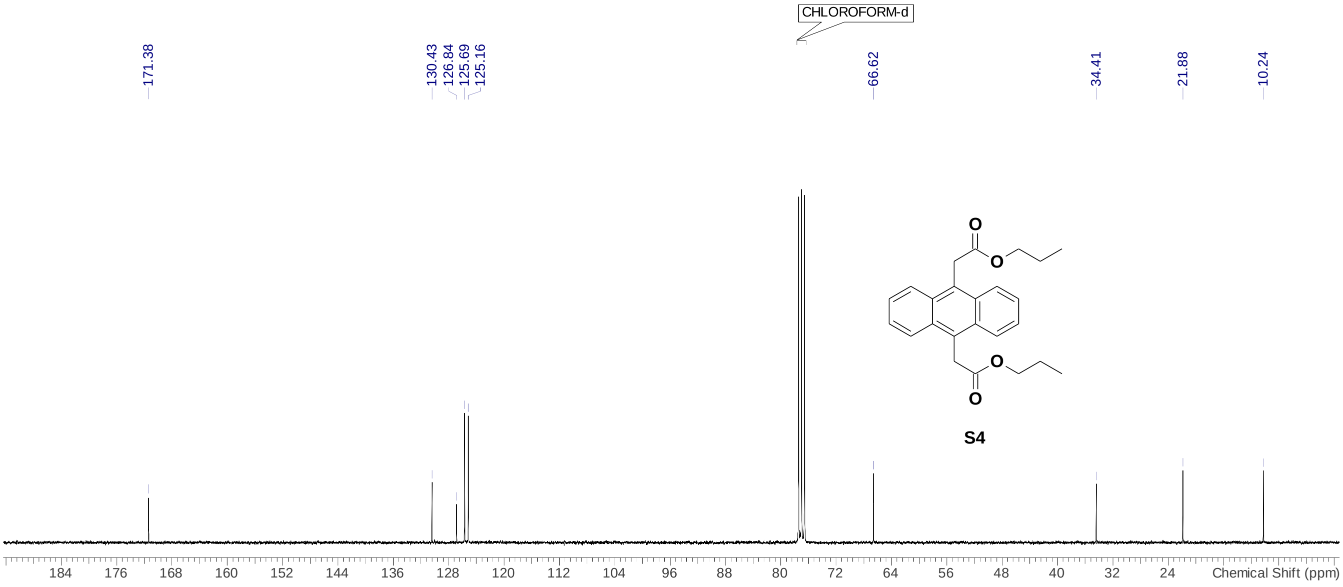

Figure S4: $^{13}C\{^{1}H\}$ NMR spectrum (75 MHz, 295 K) of **S4** in $CDCl_3$.

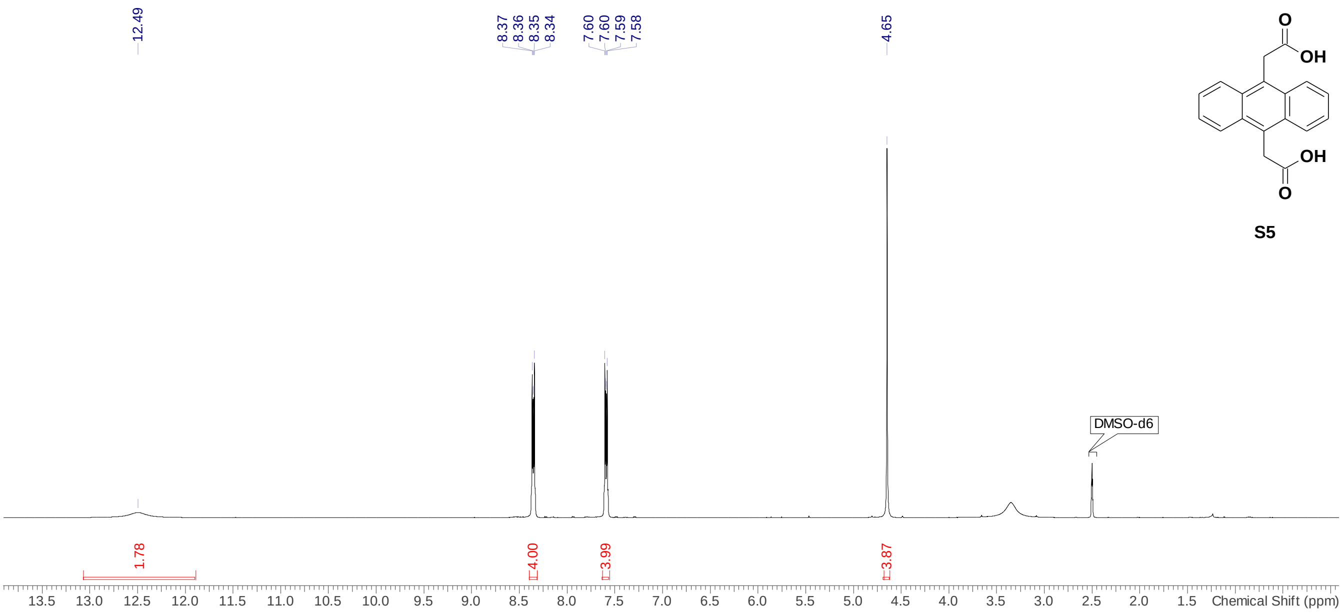

Figure S5: ^{1}H NMR spectrum (400 MHz, 295 K) of **S4** in DMSO-d_6.

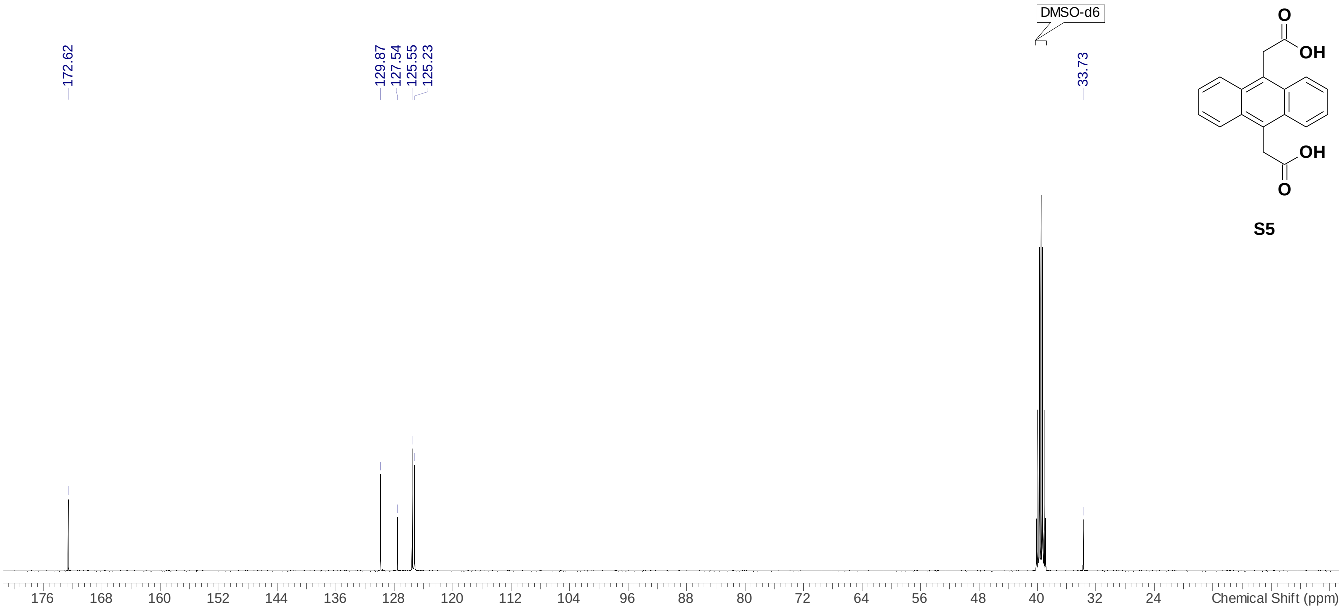

Figure S6: $^{13}C\{^{1}H\}$ NMR spectrum (100 MHz, 295 K) of **S4** in DMSO-d_6.

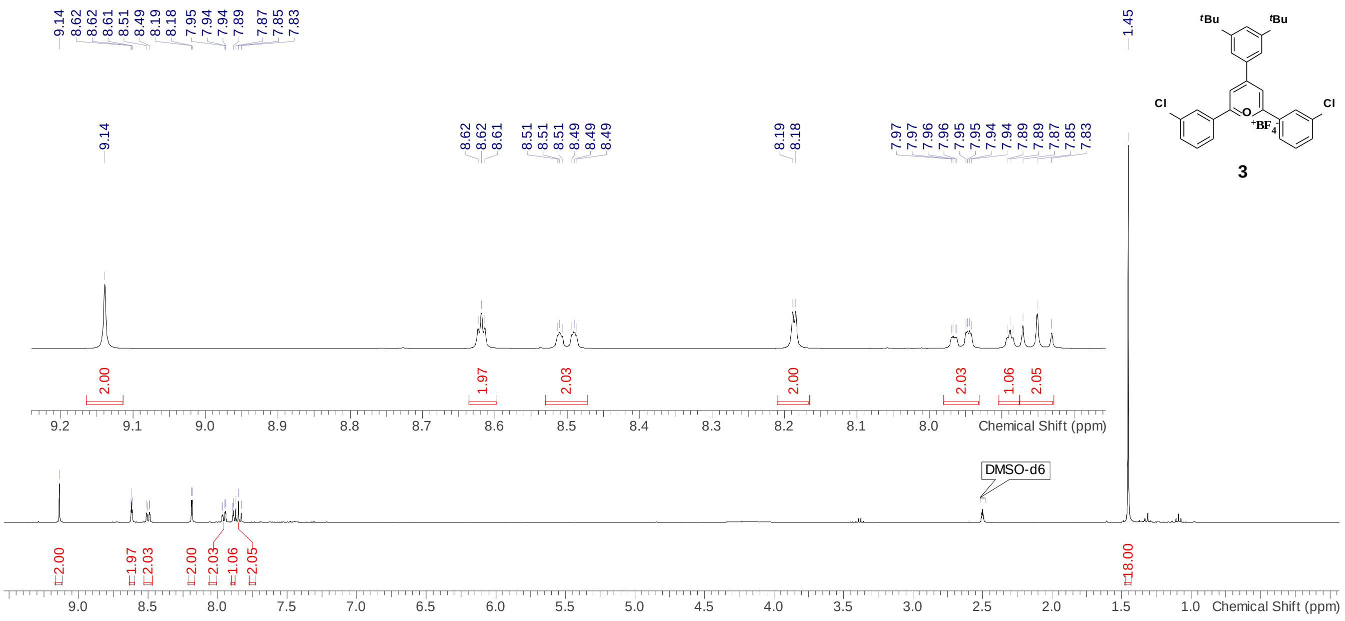

Figure S7: ^{1}H NMR spectrum (400 MHz, 295 K) of **3** in DMSO-d_6.

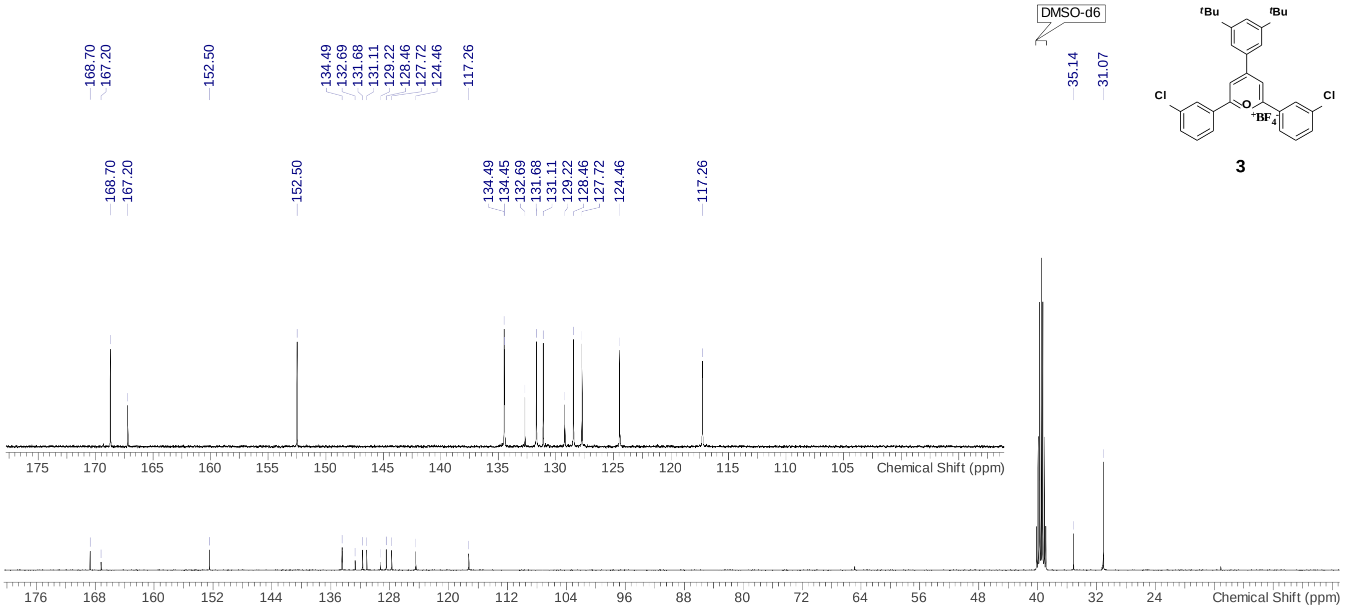

Figure S8: ^{13}C{^{1}H} NMR spectrum (100 MHz, 295 K) of **3** in DMSO-d_6.

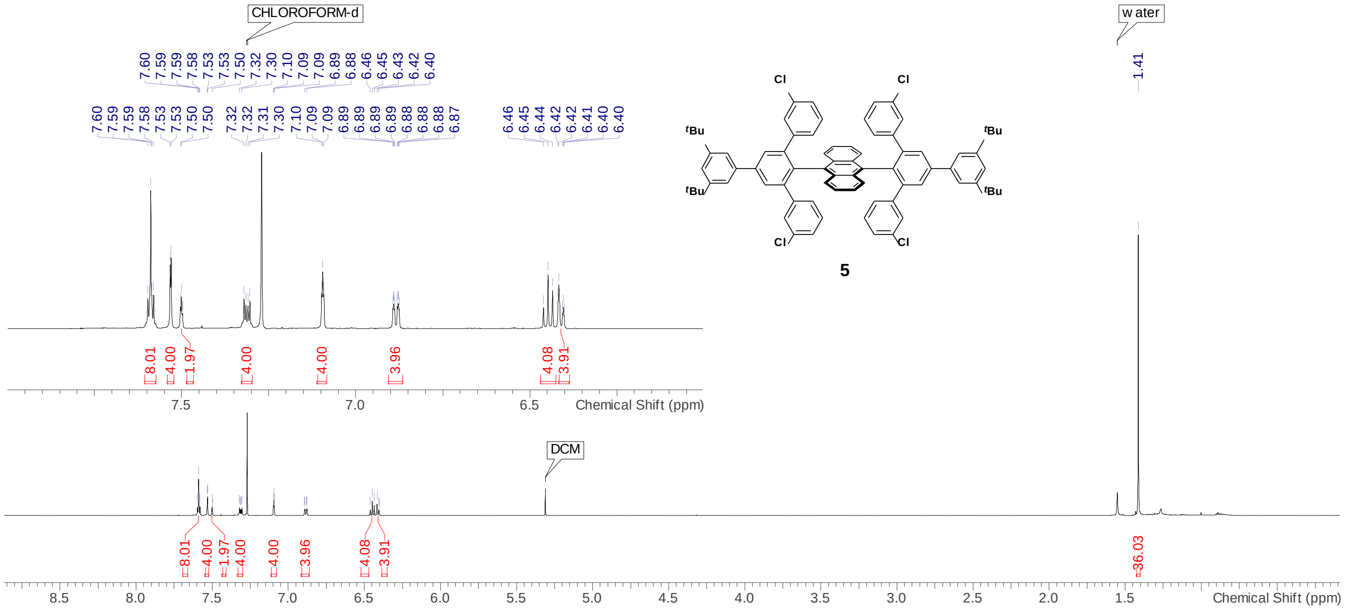

Figure S9: ^{1}H NMR spectrum (600 MHz, 295 K) of **5** in $CDCl_3$.

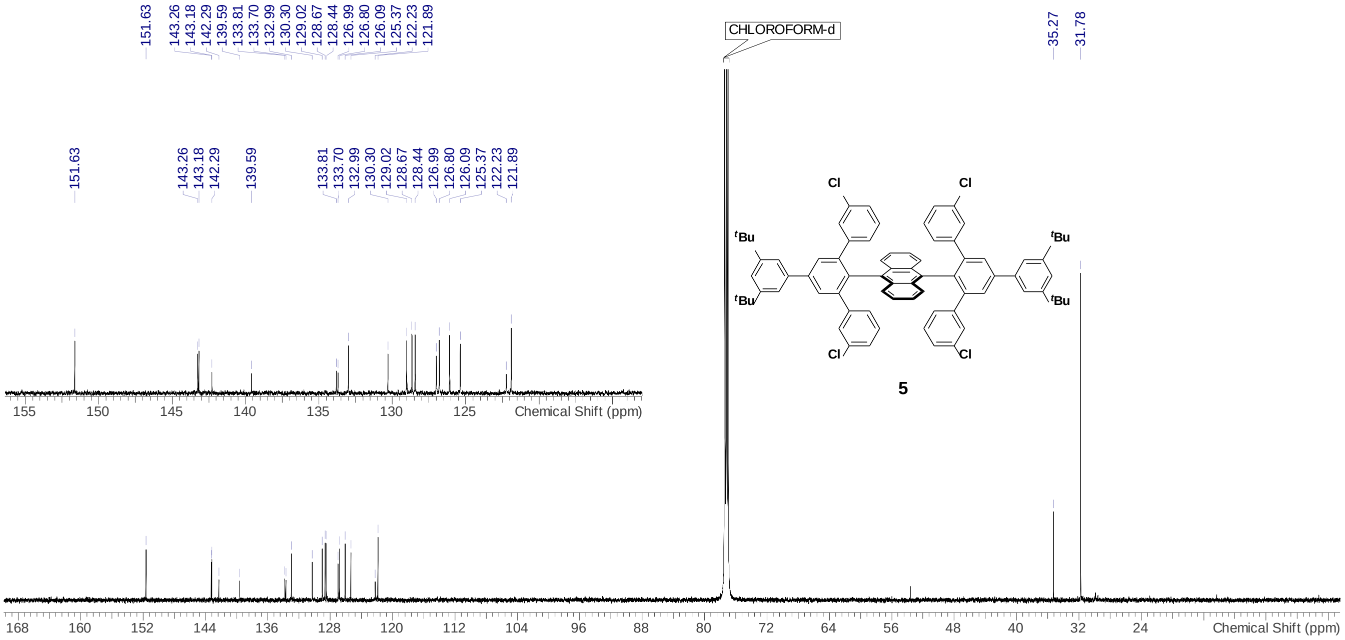

Figure S10: $^{13}C\{^1H\}$ NMR spectrum (151 MHz, 295 K) of **5** in $CDCl_3$.

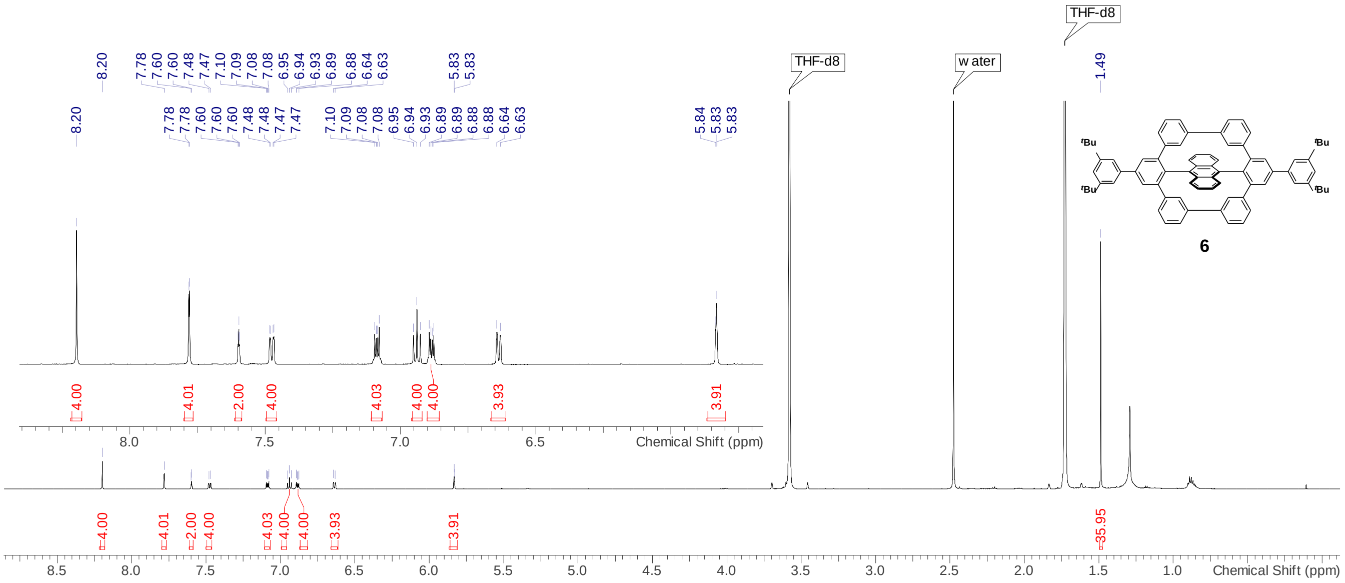

Figure S11: ^{1}H NMR spectrum (600 MHz, 295 K) of **6** in THF-d_8.

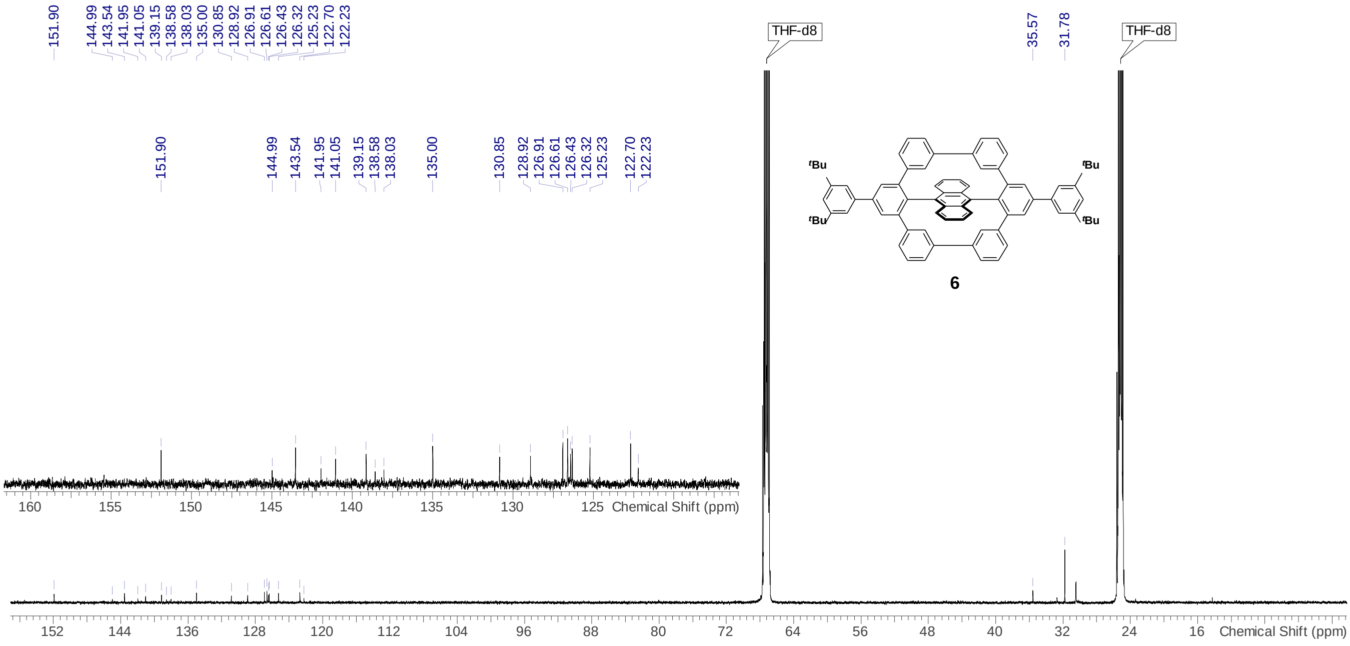

Figure S12: ^{13}C{^{1}H} NMR spectrum (151 MHz, 295 K) of **6** in THF-d_8.

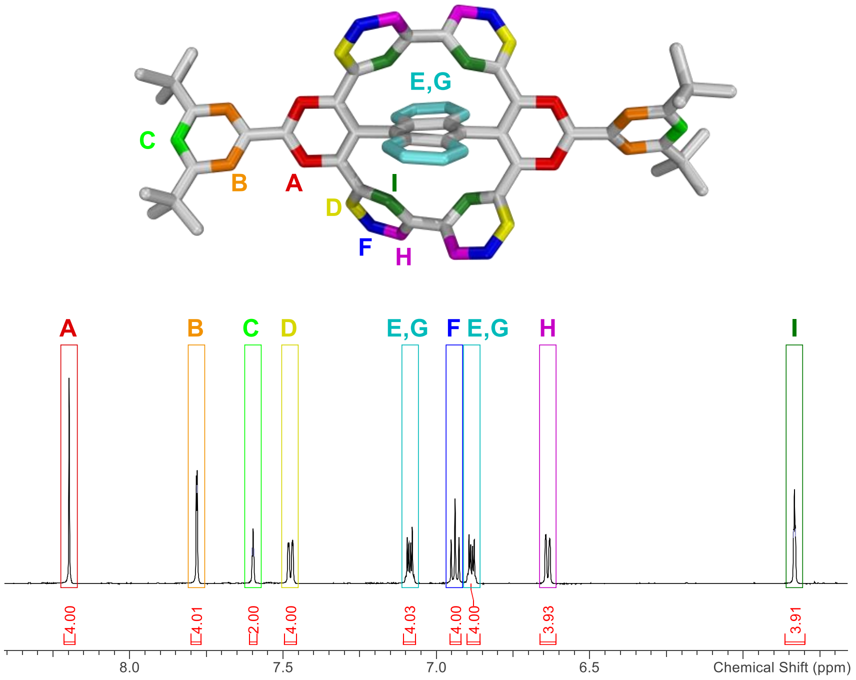

Figure S13: Assigned ^{1}H NMR spectrum (600 MHz, 295 K, THF-d_8) of **6**.

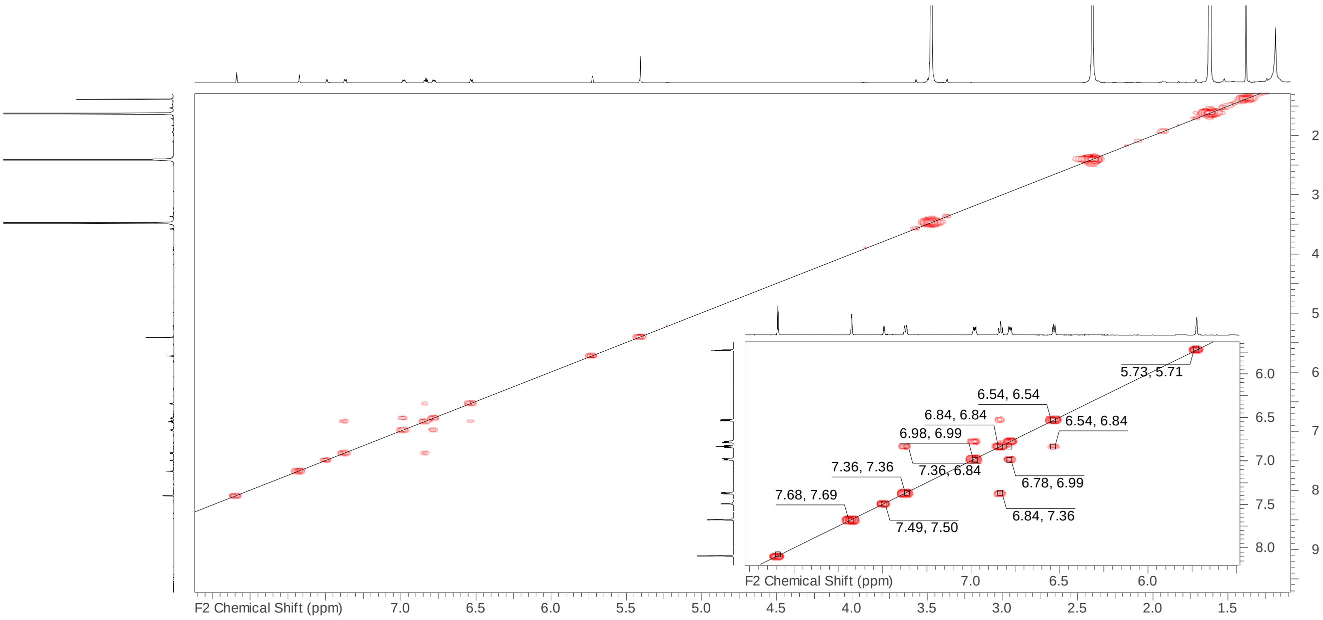

Figure S14: ^{1}H ^{1}H COSY NMR spectrum (700 MHz, 295 K) of **6** in THF-d_8.

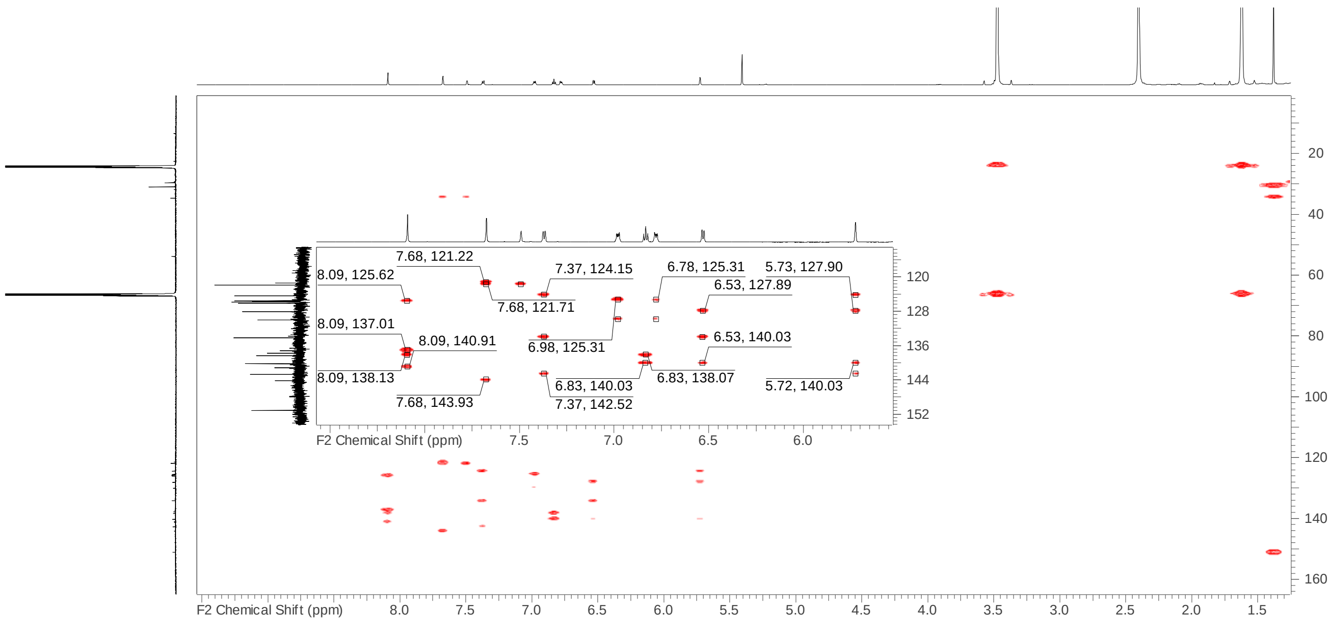

Figure S15: ^{1}H ^{13}C HSQC (me) NMR spectrum (700 MHz/176 MHz, 295 K) of **6** in THF-d8.

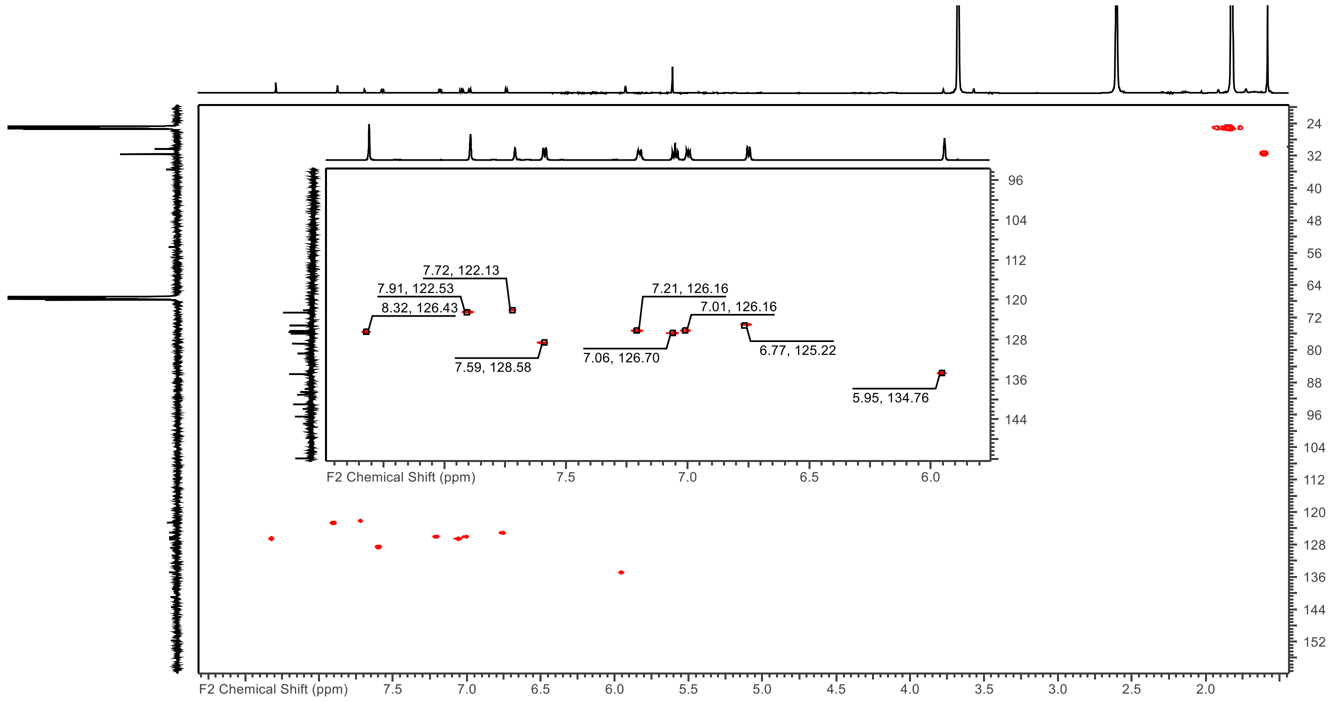

Figure S16: ^{1}H ^{13}C HMBC NMR spectrum (700 MHz/176 MHz, 295 K) of **6** in THF-d8.

2.2 Mass Analysis

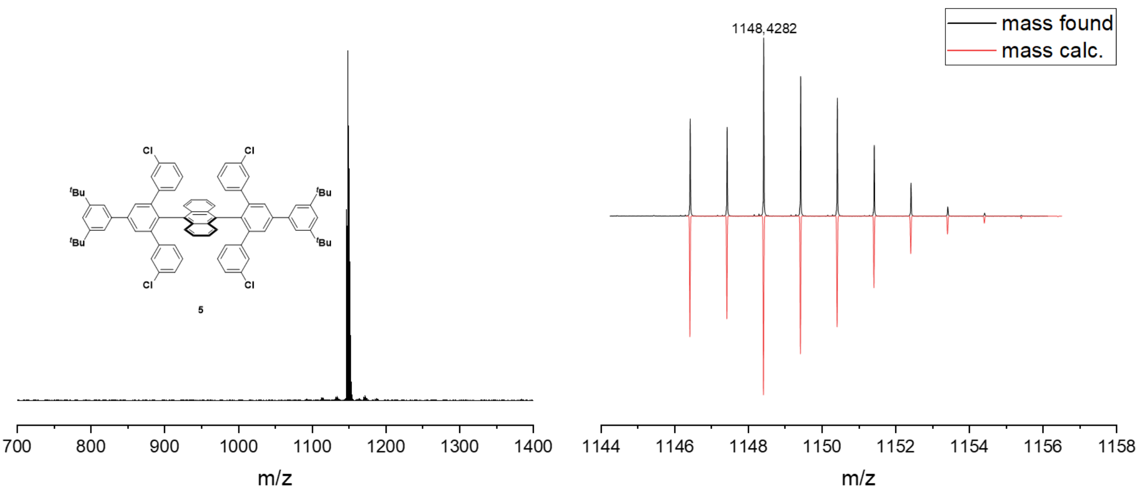

Figure S17: High resolution mass spectra (MALDI+, DCTB) of **5**.

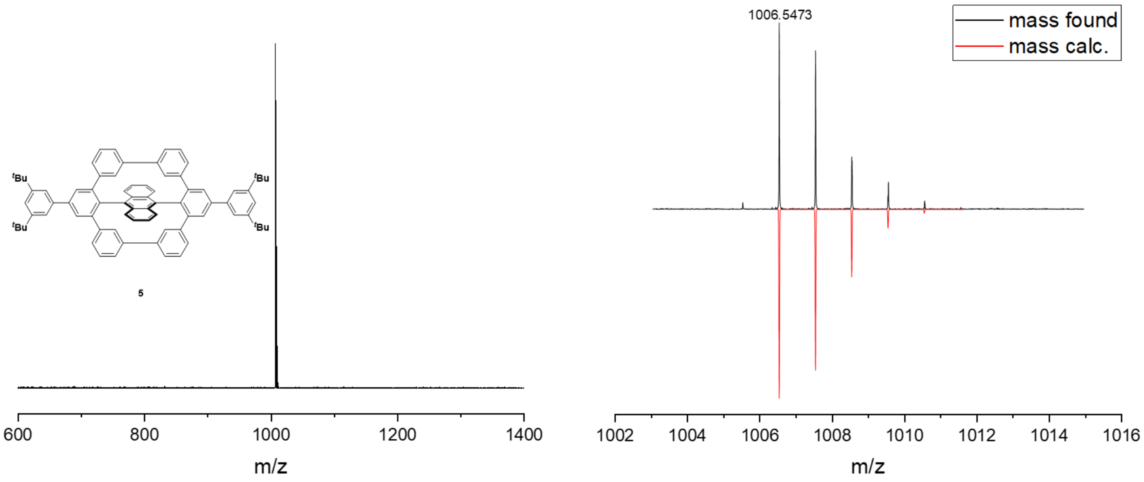

Figure S18: High resolution mass spectra (MALDI+, DCTB) of **6**.

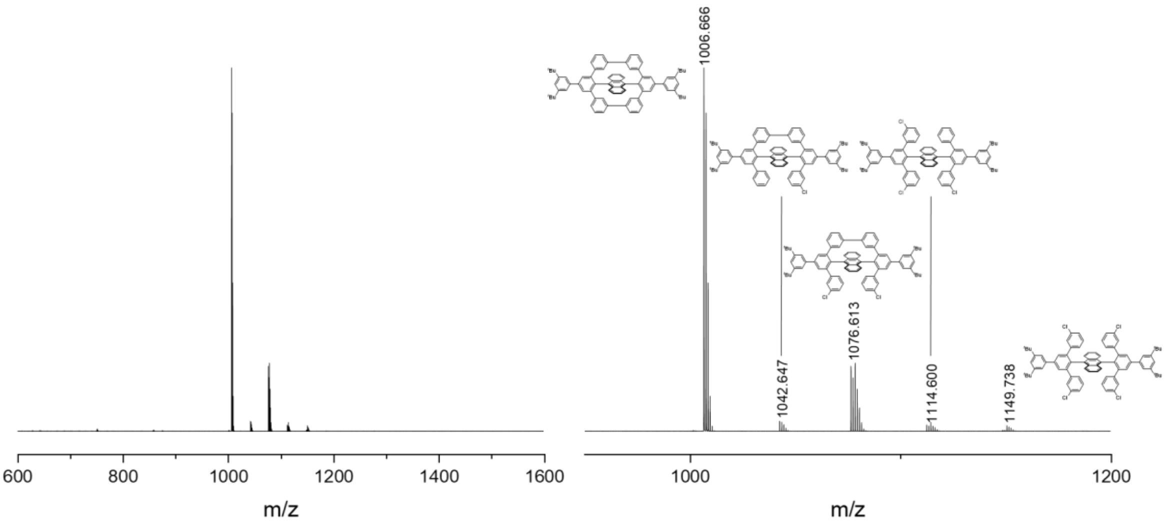

Figure S19: Mass spectra (MALDI$^+$, DCTB) of reaction mixture of **6** after 15 min reaction time, spectra overview (left) and identified side products (right).

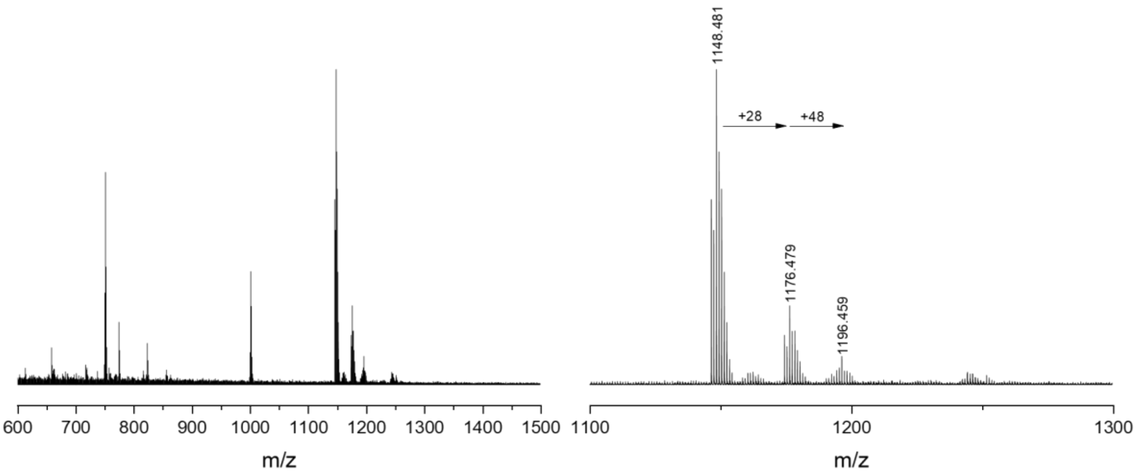

Figure S20: Mass spectra (MALDI$^+$, DCTB) of **5** after irradiation with a handheld UV-lamp (λ_1= 254 nm and λ_2= 365 nm) over 12 h in dichloromethane under ambient conditions.

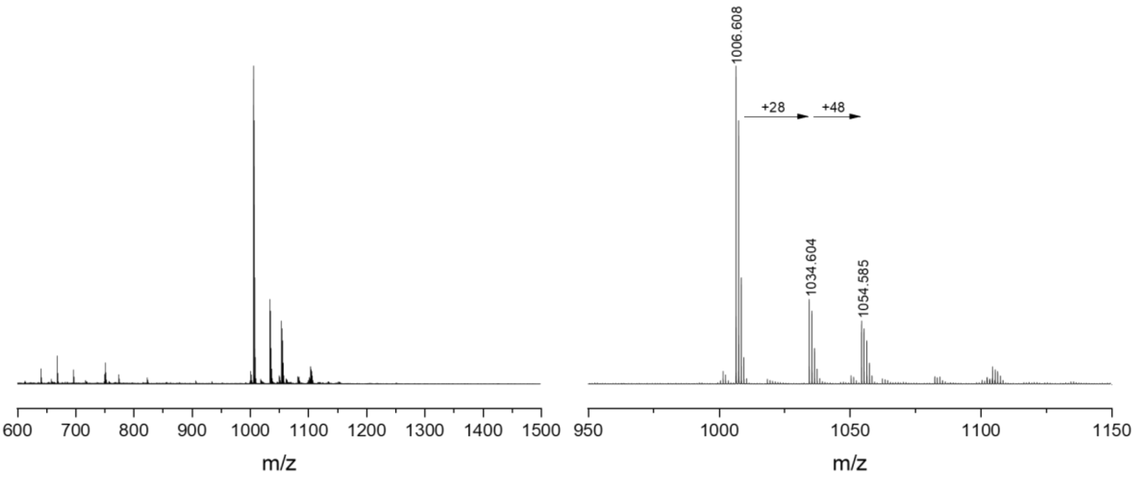

Figure S21: Mass spectra (MALDI$^+$, DCTB) of **6** after irradiation with a handheld UV-lamp (λ_1= 254 nm and λ_2= 365 nm) over 12 h in dichloromethane under ambient conditions.

2.3 Stability Measurements

2.3.1 Stability Measurements in Cyclohexane

All UV-vis stability studies were performed irradiating dilute solutions (10^{-5}mol/L in 3.00 mL cyclohexane (HPLC grade)) of the sample in a quartz cuvette at room temperature. Irradiation of all samples was performed in a Rayonet RPR-200 photochemical reactor (16 lamps, 254 nm). All samples were placed in the middle of the photoreactor with a distance of 10 cm to the lamps. Measurements under ambient conditions were performed under air atmosphere while measurements under argon atmosphere were performed in flame dried inert gas quartz cuvettes. For measurements under argon atmosphere the solvent was degassed for 2 h by bubbling an argon stream through the solution (in a flame dried schlenk flask).

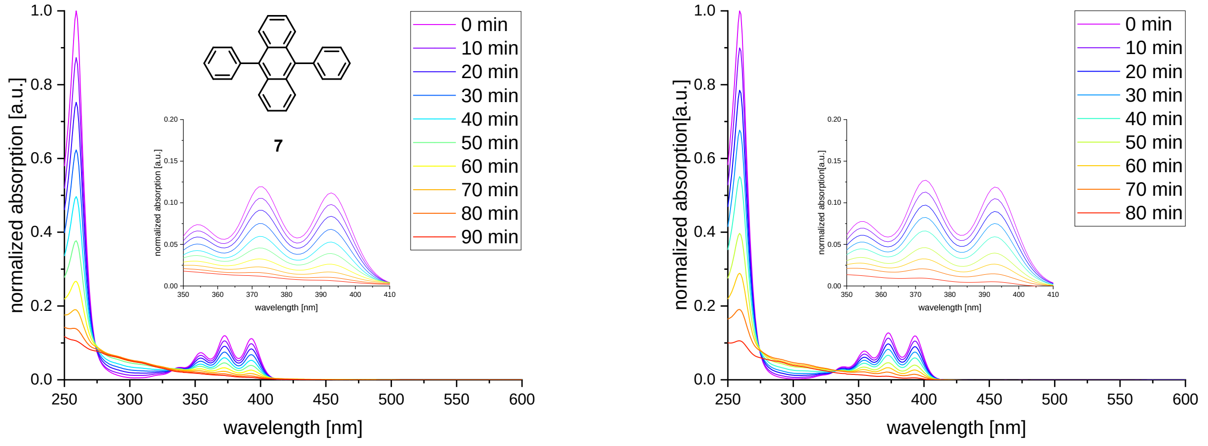

Figure S22: Normalized change in absorption intensity of a solution of **7** while irradiating in cyclohexane under ambient conditions (left) and argon atmosphere (right).

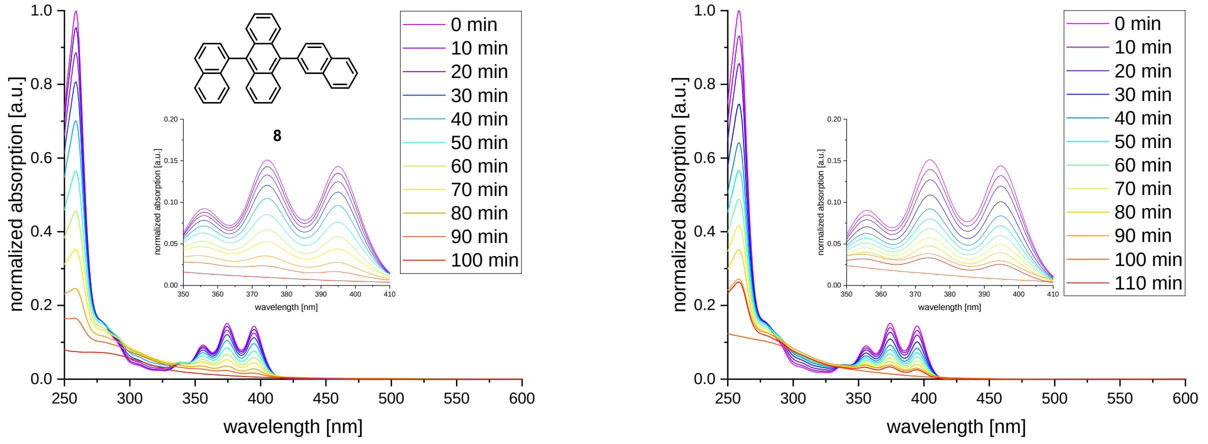

Figure S23: Normalized change in absorption intensity of a solution of **8** while irradiating in cyclohexane under ambient conditions (left) and argon atmosphere (right).

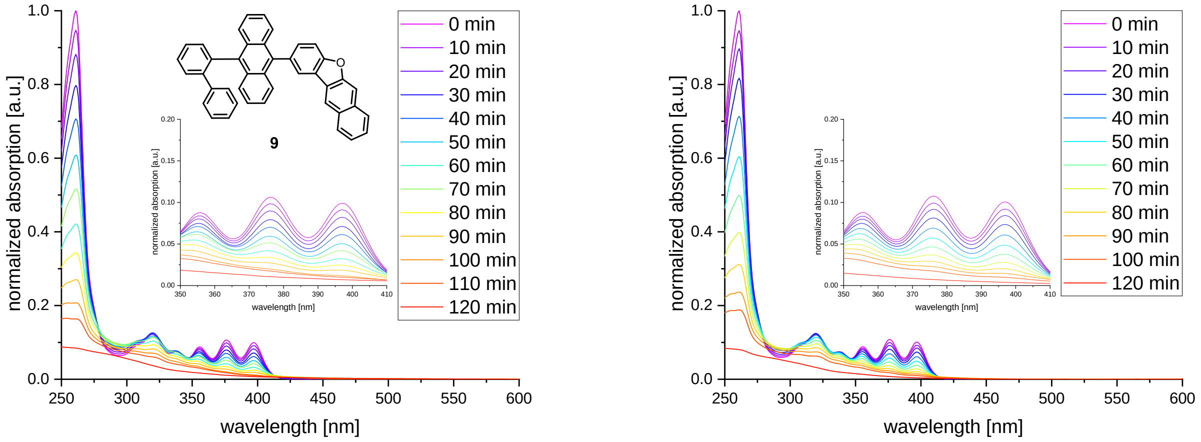

Figure S24: Normalized change in absorption intensity of a solution of **9** while irradiating in cyclohexane under ambient conditions (left) and argon atmosphere (right).

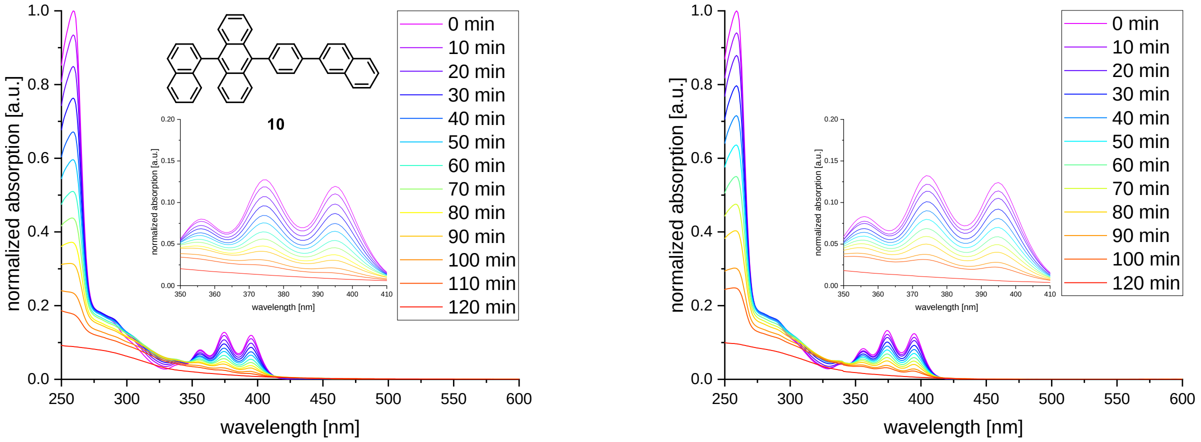

Figure S25: Normalized change in absorption intensity of a solution of **10** while irradiating in cyclohexane under ambient conditions (left) and argon atmosphere (right).

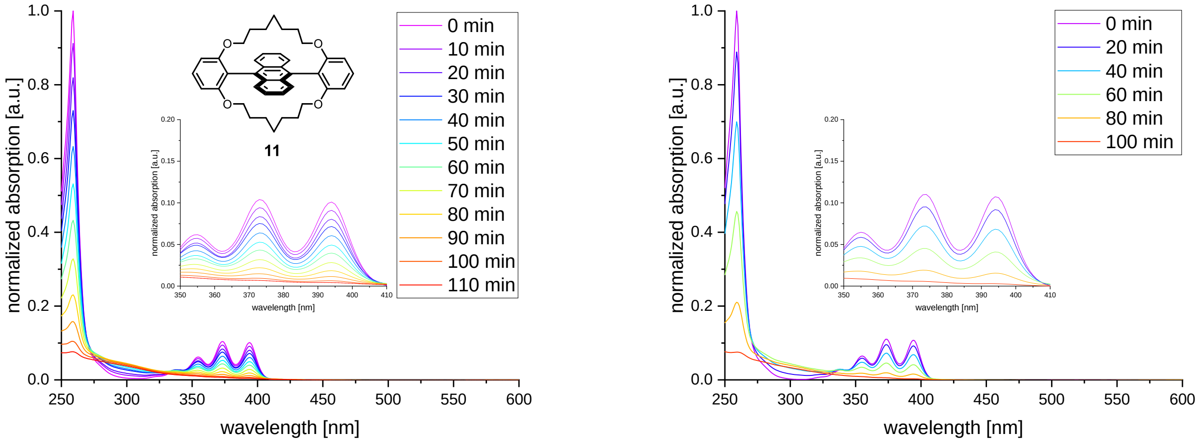

Figure S26: Normalized change in absorption intensity of a solution of **11** while irradiating in cyclohexane under ambient conditions (left) and argon atmosphere (right).

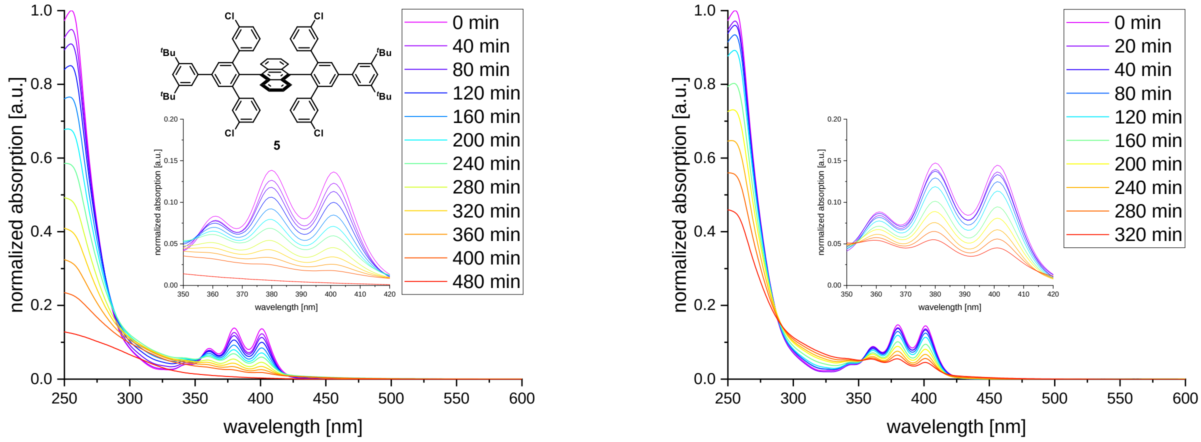

Figure S27: Normalized change in absorption intensity of a solution of **5** while irradiating in cyclohexane under ambient conditions (left) and argon atmosphere (right).

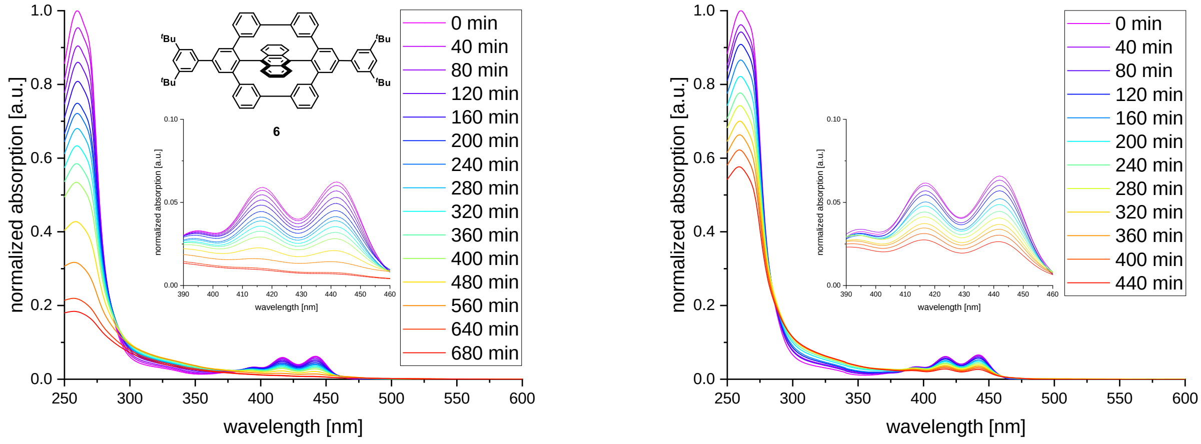

Figure S28: Normalized change in absorption intensity of a solution of **6** while irradiating in cyclohexane under ambient conditions (left) and argon atmosphere (right).

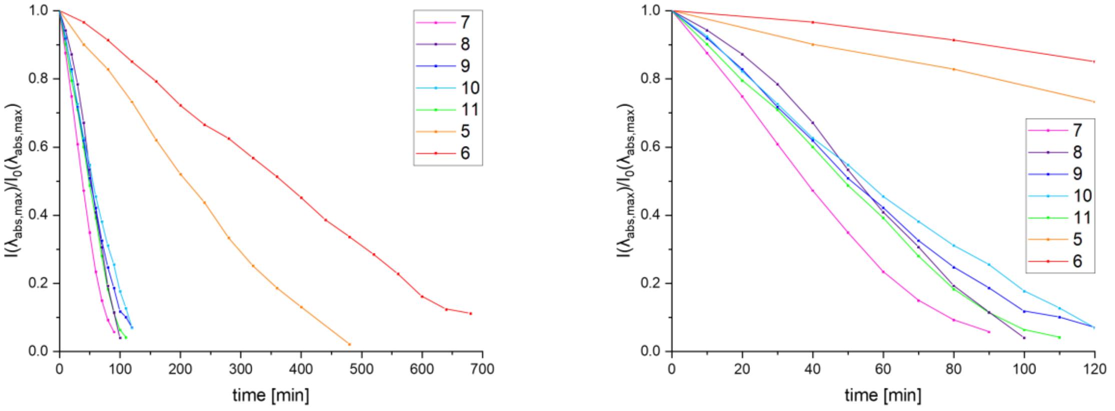

Figure S29: Time dependent evolution of absorption intensity at $\lambda_{abs,\ max}$ for **5**-**11** while irradiating in cyclohexane under ambient conditions (left) and magnified section of the plot to show half-lives of **7**-**11** (right).

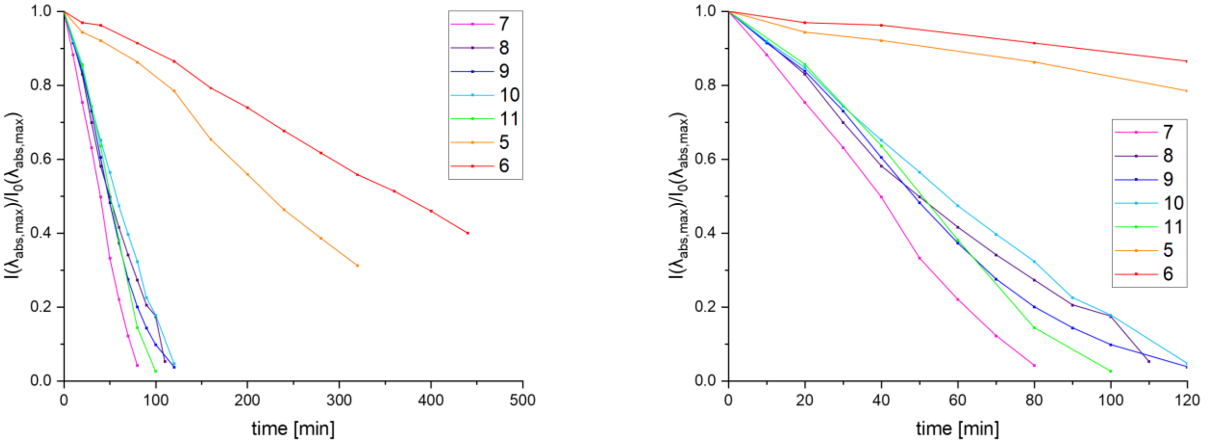

Figure S30: Time dependent evolution of absorption intensity at $\lambda_{abs,\ max}$ for **5**-**11** while irradiating in cyclohexane under argon atmosphere (left) and magnified section of the plot to show half-lives of **7**-**11** (right).

2.3.2 Stability Measurements in Dichloromethane

All UV-vis stability studies were performed irradiating dilute solutions (10^{-5}mol/L in 3.00 mL dichloromethane (HPLC grade)) of the sample in a quartz cuvette at room temperature. Irradiation of all samples was performed using a handheld UV-lamp from HEROLAB GmbH LABORGERÄTE, Wiesloch (type: NU-15, λ_1= 254 nm (15W) and λ_2= 365 nm (15W)). All samples were irradiated with 254 nm and 365 nm at the same time with a distance of 12 cm to the lamp. Measurements under ambient conditions were performed under air atmosphere while measurements under argon atmosphere were performed in flame dried inert gas quartz cuvettes. For measurements under argon atmosphere the solvent was degassed for 2 h by bubbling an argon stream through the solution (in a flame dried schlenk flask).

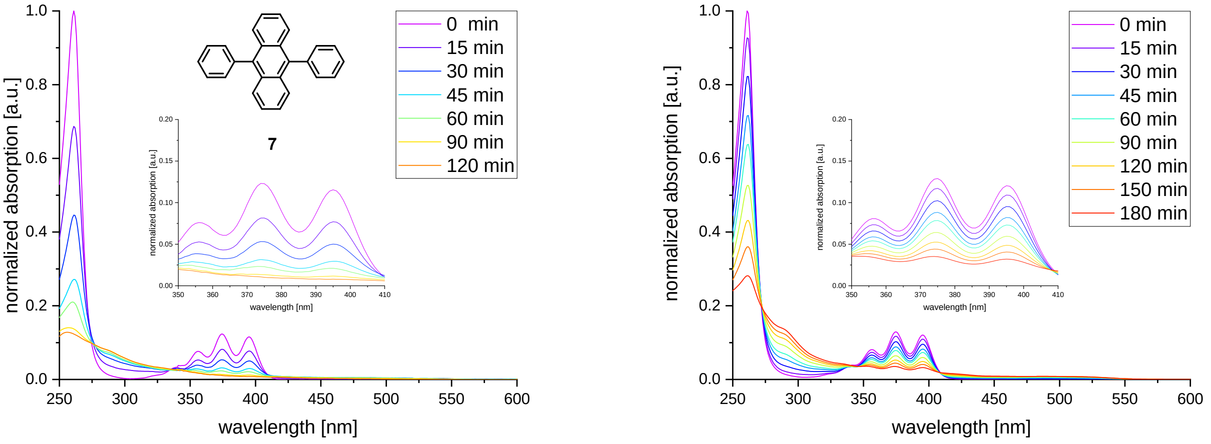

Figure S31: Normalized change in absorption intensity of a solution of **7** while irradiating in dichloromethane under ambient conditions (left) and argon atmosphere (right).

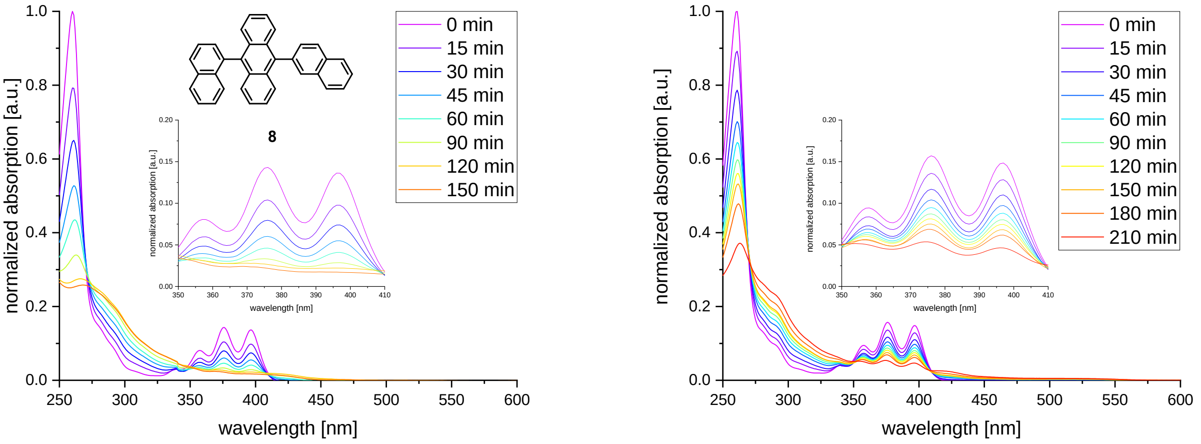

Figure S32: Normalized change in absorption intensity of a solution of **8** while irradiating in dichloromethane under ambient conditions (left) and argon atmosphere (right).

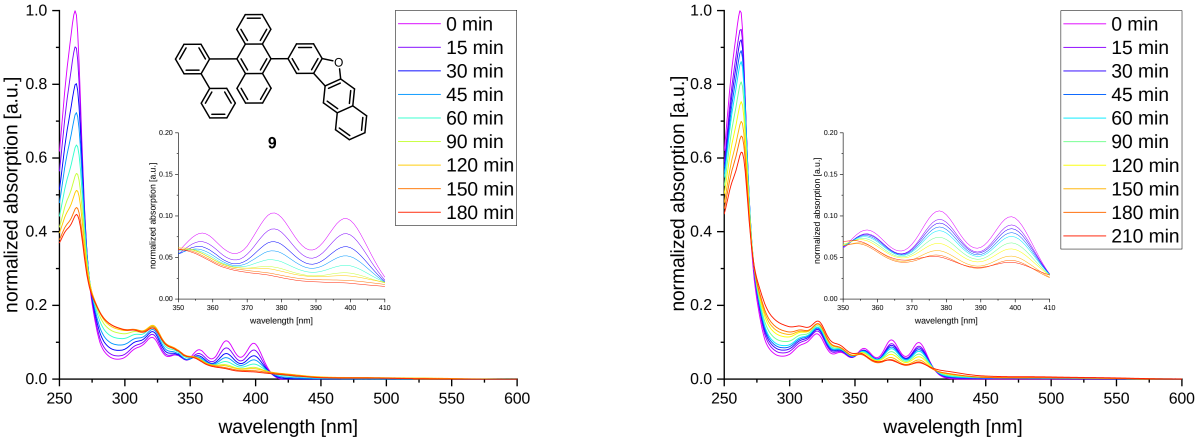

Figure S33: Normalized change in absorption intensity of a solution of **9** while irradiating in dichloromethane under ambient conditions (left) and argon atmosphere (right).

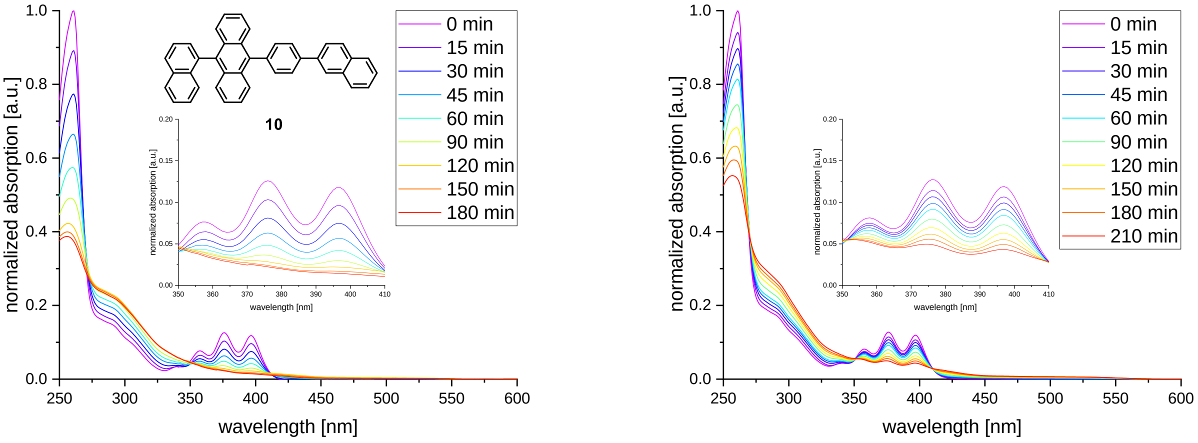

Figure S34: Normalized change in absorption intensity of a solution of **10** while irradiating in dichloromethane under ambient conditions (left) and argon atmosphere (right).

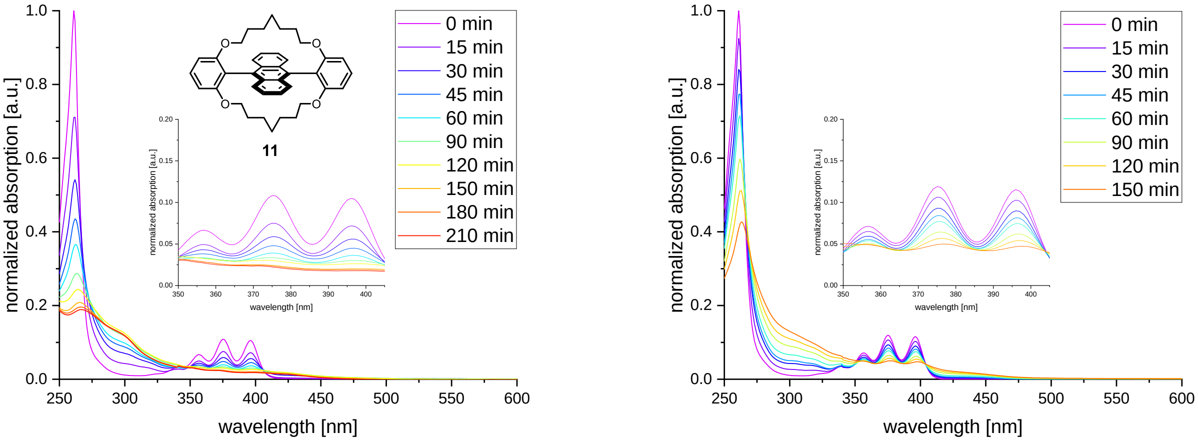

Figure S35: Normalized change in absorption intensity of a solution of **11** while irradiating in dichloromethane under ambient conditions (left) and argon atmosphere (right).

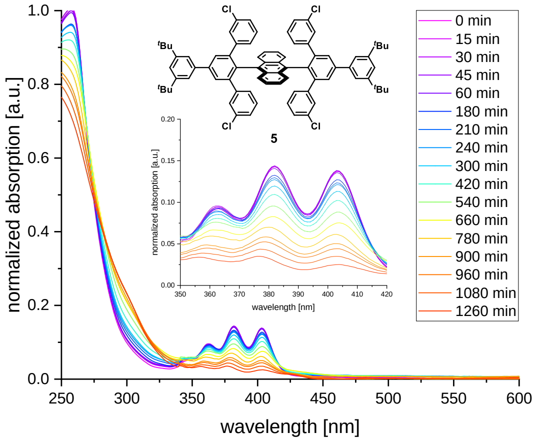

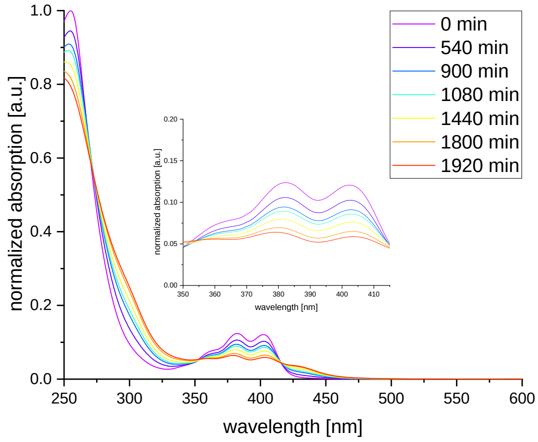

Figure S36: Normalized change in absorption intensity of a solution of **5** while irradiating in dichloromethane under ambient conditions (left) and argon atmosphere (right).

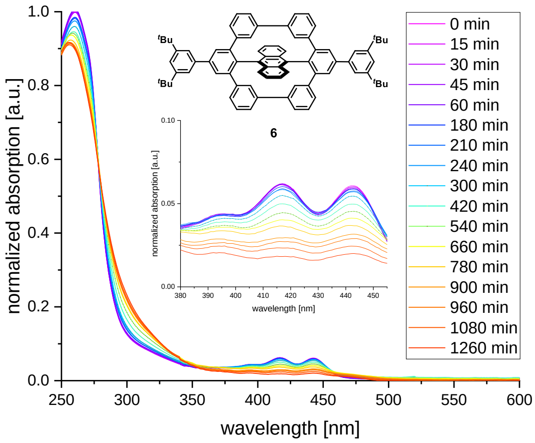

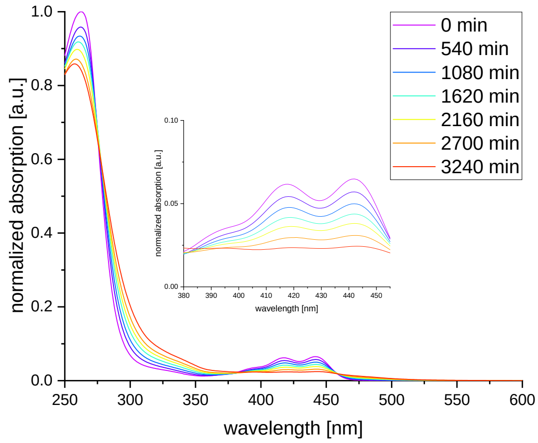

Figure S37: Normalized change in absorption intensity of a solution of **6** while irradiating in dichloromethane under ambient conditions (left) and argon atmosphere (right).

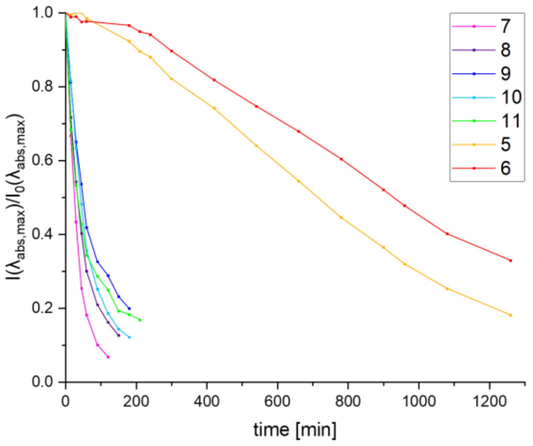

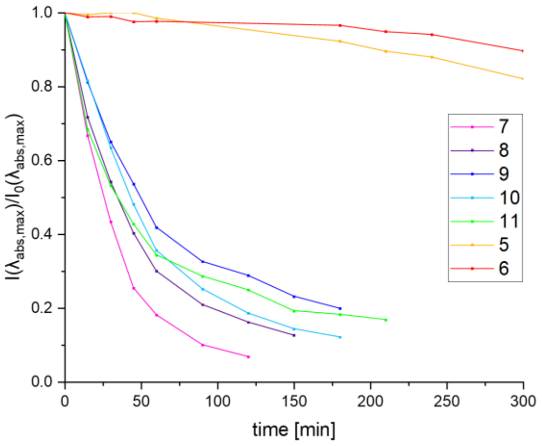

Figure S38: Time dependent evolution of absorption intensity at $\lambda_{abs,\ max}$ for **5**-**11** while irradiation in dichloromethane under ambient conditions (left) and magnified section of the plot to show half-lives of **7**-**11** (right).

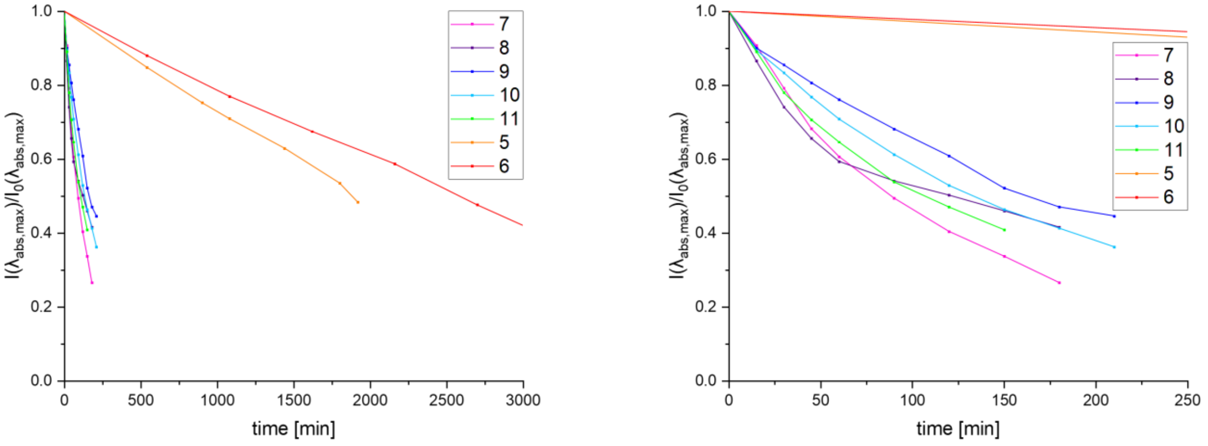

Figure S39: Time dependent evolution of absorption intensity at $\lambda_{abs,\ max}$ for **5**-**11** while irradiation in dichloromethane under argon atmosphere (left) and magnified section of the plot to show half-lives of **7**-**11** (right).

2.3.3 Stability Measurements of thin films

All UV-vis stability studies were performed irradiating spin-coated films (10 mg/ 3 mL, chloroform, 1000 rpm, 40 s, substrate: Menzel microscope slides) at room temperature. Irradiation of all films was performed in a Rayonet RPR-200 photochemical reactor (16 lamps, 254 nm). All films were placed in the middle of the photoreactor with a distance of 10 cm to the lamps. All measurements were performed under ambient conditions. Films of **7** and **11** were omitted in our comparison as they were crystalline (with amorphous regions) after decomposition (as evidenced by polarized light microscopy images, see section 2.4). This resulted in scattering and increased stability compared to amorphous films of **8**-**10** and **5**. A measurement of **6** was not successful as all spin-coating attempts resulted in too thin films for absorption analysis. Attempts to increase film thicknesses by decreasing the spin-coating speed to 250-750 rpm or via increased concentration of **6** in chloroform (20 mg/ 3 mL) failed. Attempts to increase the film thickness by drop casting led to crystalline films resulting in highly increased stability. Note that for measurements in cyclohexane and DCM, the stability of **6** was slightly increased compared to **5**, which may also be the case in thin films. However, the increased stability of **5** compared to **8**-**10** in thin films underlines the importance of *meta*-terphenylyl-protected anthracenes.

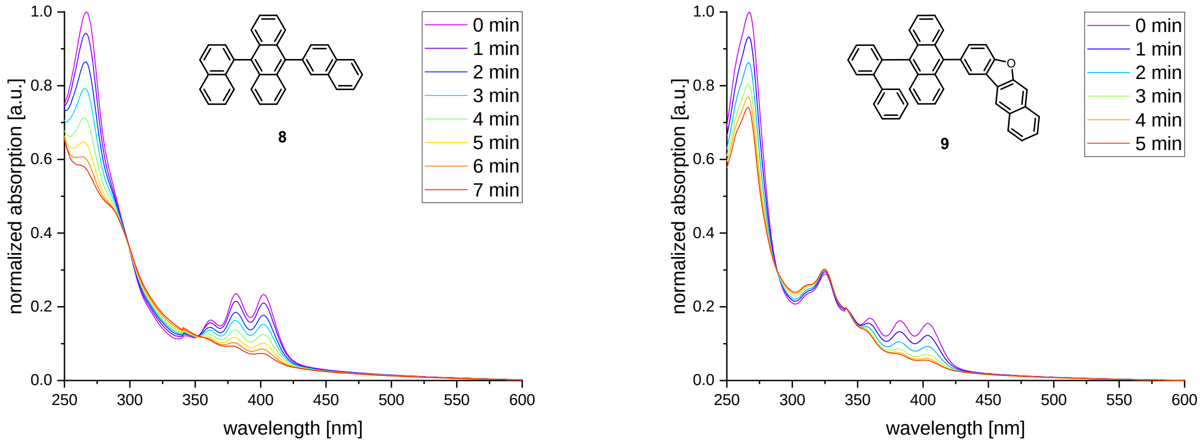

Figure S40: Normalized change in absorption intensity of a thin film of **8** (left) and **9** (right) while irradiating under ambient conditions.

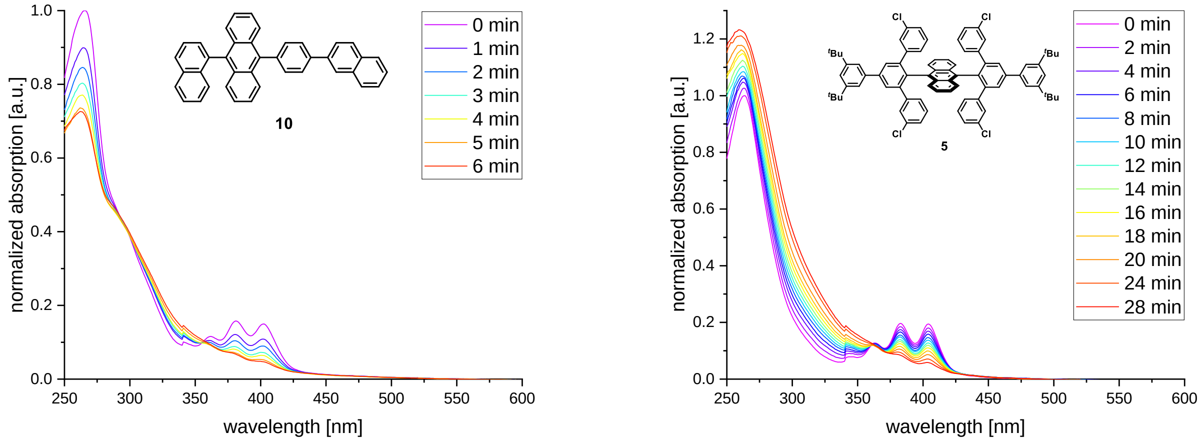

Figure S41: Normalized change in absorption intensity of a thin film of **10** (left) and **5** (right) while irradiating under ambient conditions.

2.4 Polarized Light Micrographs

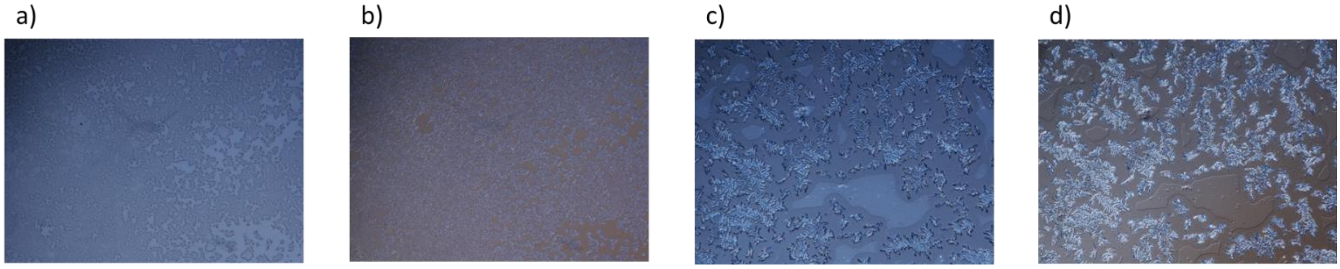

Figure S42: Polarized light microscopy images of **7**: a) 20x zoom (light), b) 20x zoom (polarized light), c) 100x zoom (light) and d) 100x zoom (polarized light).

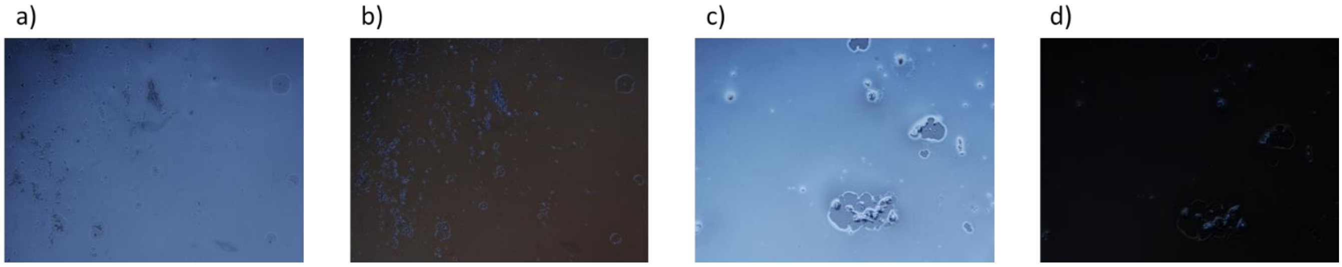

Figure S43: Polarized light microscopy images of **8**: a) 20x zoom (light), b) 20x zoom (polarized light), c) 100x zoom (light) and d) 100x zoom (polarized light).

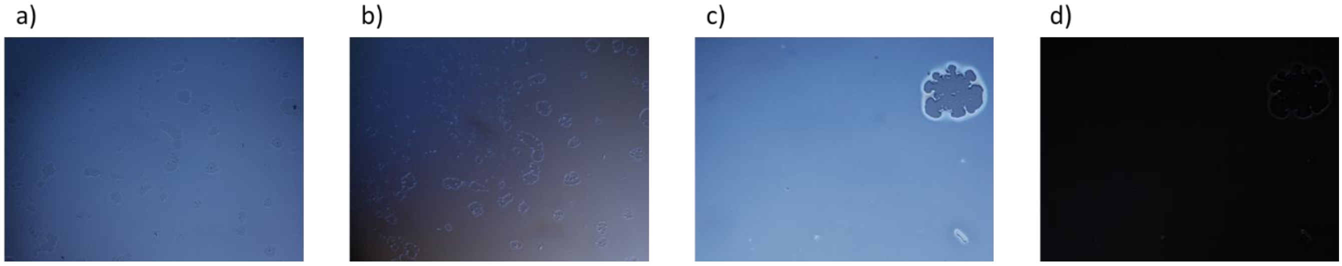

Figure S44: Polarized light microscopy images of **9**: a) 20x zoom (light), b) 20x zoom (polarized light), c) 100x zoom (light) and d) 100x zoom (polarized light).

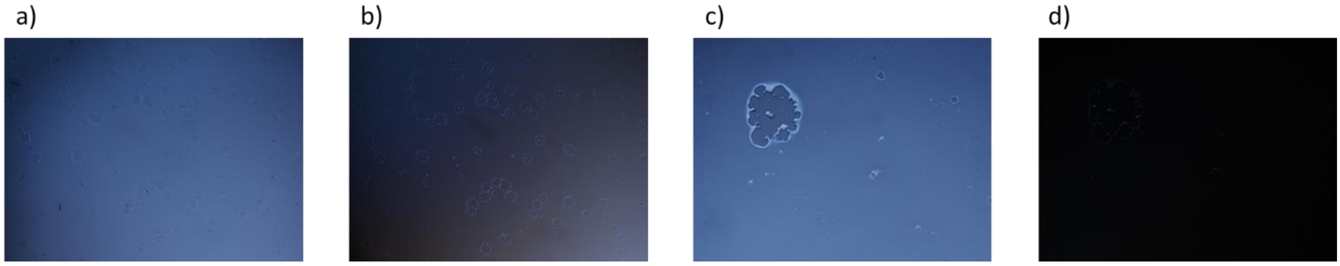

Figure S45: Polarized light microscopy images of **10**: a) 20x zoom (light), b) 20x zoom (polarized light), c) 100x zoom (light) and d) 100x zoom (polarized light).

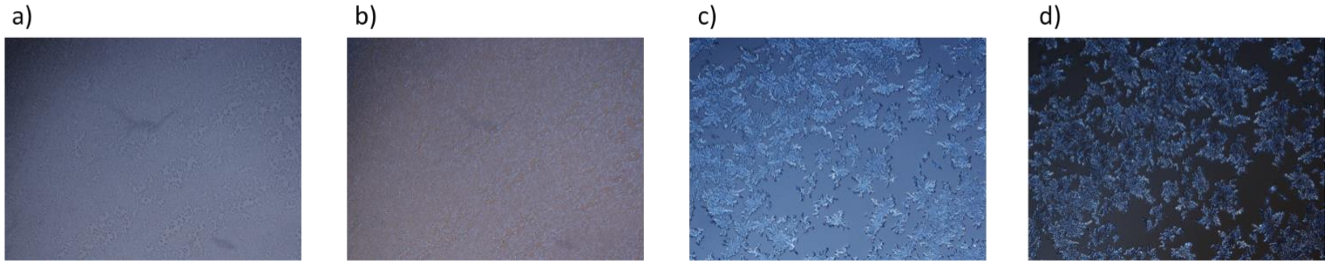

Figure S46: Polarized light microscopy images of **11**: a) 20x zoom (light), b) 20x zoom (polarized light), c) 100x zoom (light) and d) 100x zoom (polarized light).

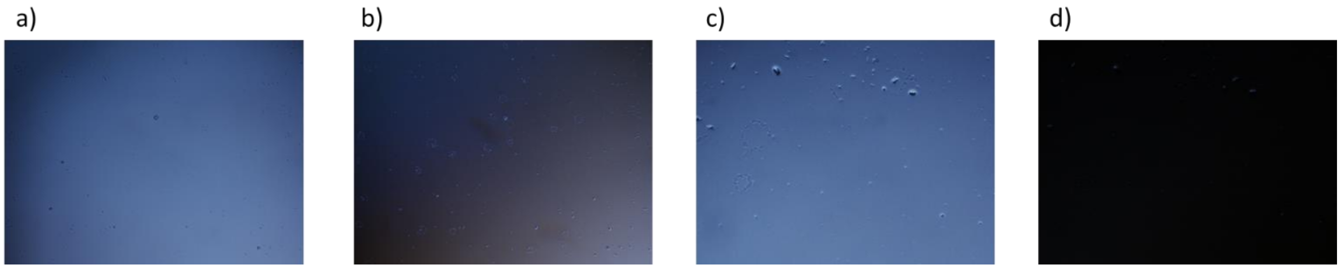

Figure S47: Polarized light microscopy images of **5**: a) 20x zoom (light), b) 20x zoom (polarized light), c) 100x zoom (light) and d) 100x zoom (polarized light).

2.5 Calculations

All calculations were performed using Gaussian16.[S5] At first, the gas-phase ground-state equilibrium geometry of the molecules was optimized at the B3LYP/def2-SVP level of theory. The received geometries were further refined using the B3LYP/def2-TZVP level of theory. FMO calculations were performed using the optimized geometries on the B3LYP/def2-TZVP level of theory.

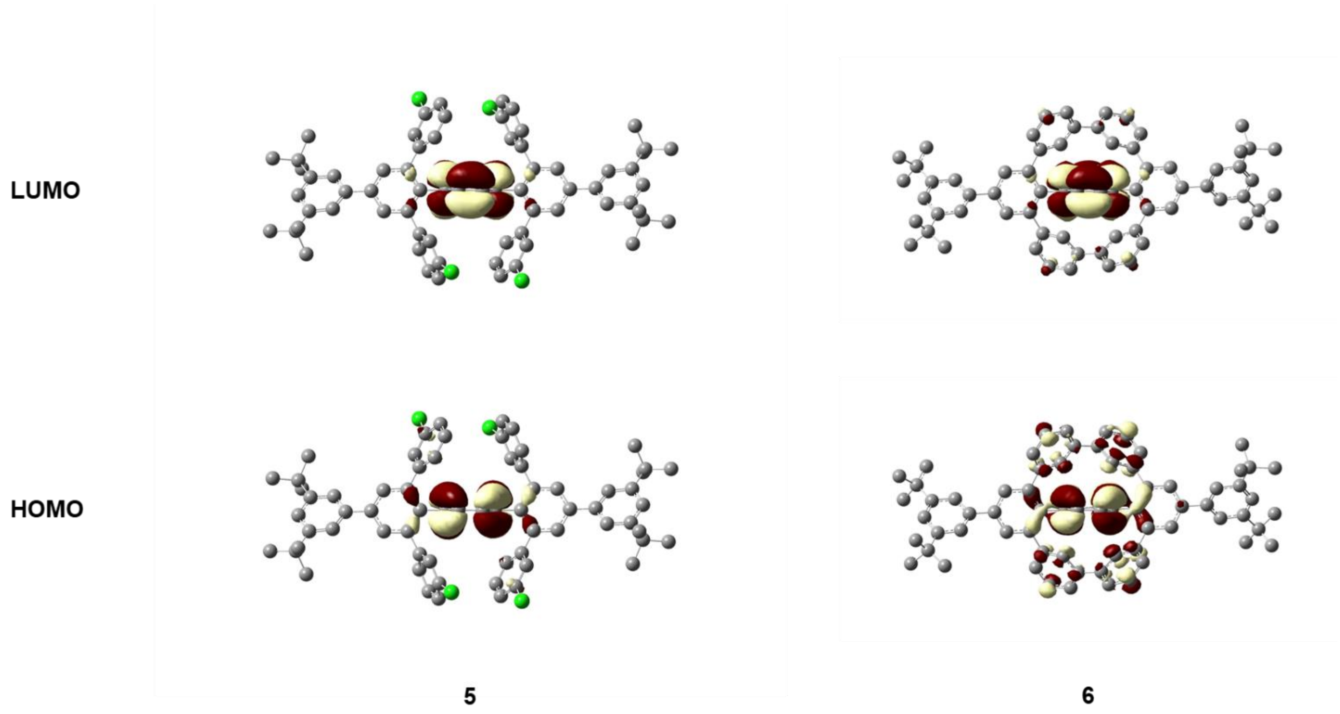

Figure S48: Calculated FMO distribution of **5** (left) and **6** (right), side view.

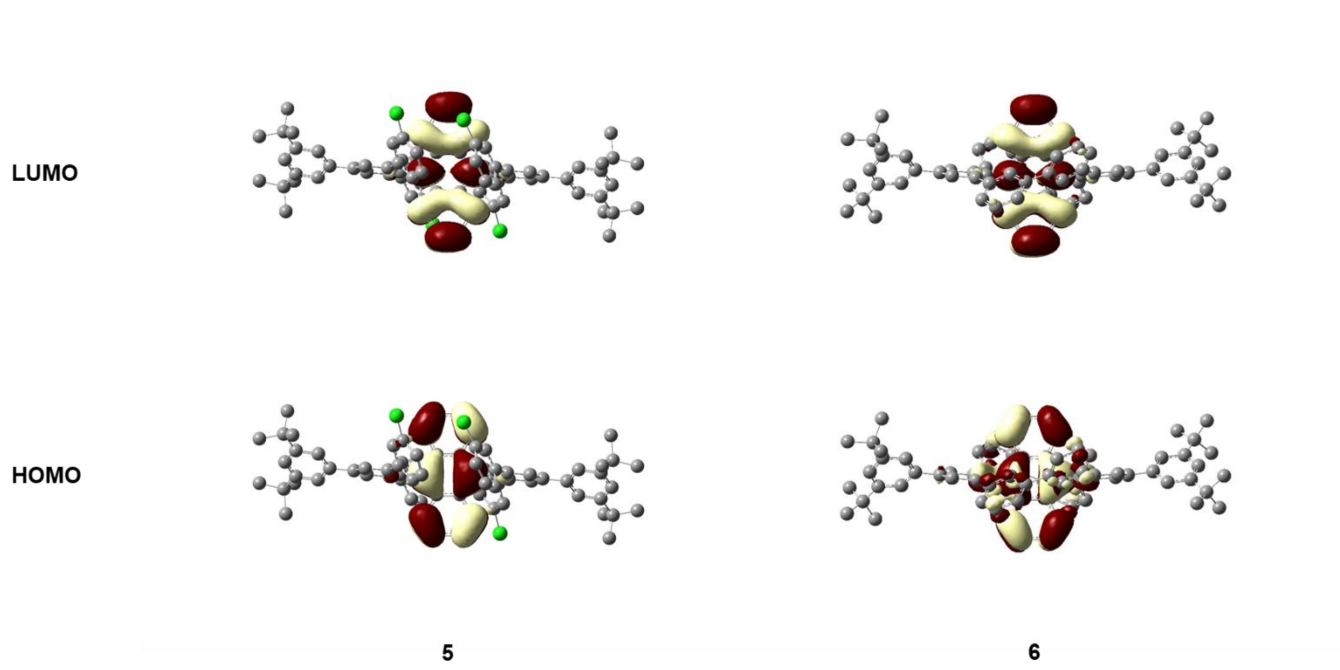

Figure S49: Calculated FMO distribution of **5** (left) and **6** (right), top view.

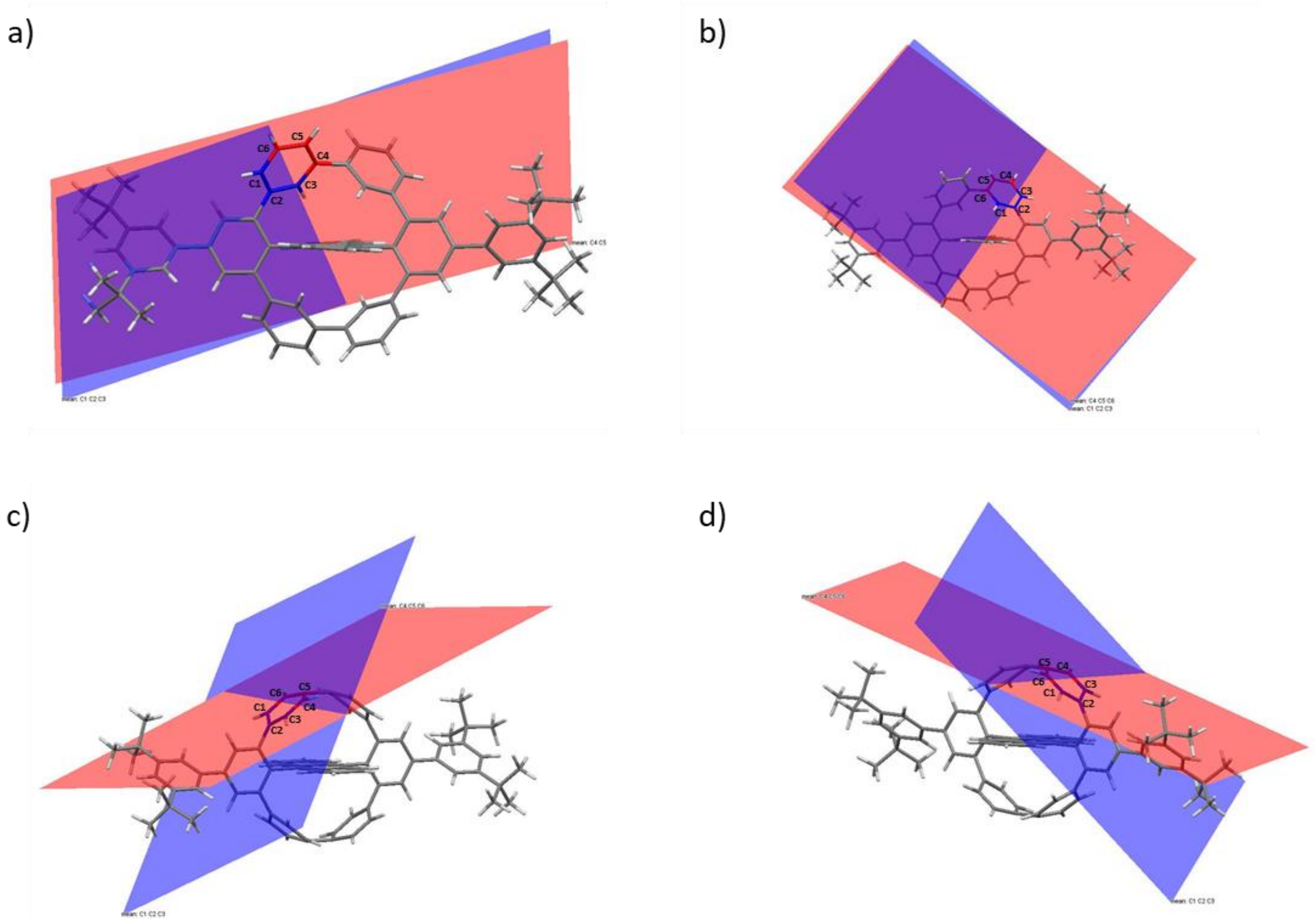

Figure S50: Visualization of the bending of the bridging benzene rings in [6]cyclo-meta-hexaphenylene-wrapped anthracene **6** (a,b) and *para*-analogue by Höger *et al.*(c,d). For calculation of the bending angle of the bridging benzene rings two planes were put through C1, C2, C3 (plane 1) and C4, C5, C6 (plane 2) and the angle between the two planes was calculated. For **6** the bend angles were determined to be 6.03° (a, left benzene ring) and 5.23° (b, right benzene ring), while the Höger analogue showed a much higher bend angle of 32.32° (c+d). For plane calculations the geometry optimized structures of both molecules (Gaussian16, B3LYP/def2SVP/B3LYP/def2TZVP)[S5] were used. Additional to the bend angles the steric energy of both molecules was calculated by two methods: 1) The steric energy was calculated by a geometry optimization (MM2 calculation) using Chem3D for both molecules, 2) The ground state energies of both molecules were calculated using (Gaussian16, B3LYP/def2SVP/B3LYP/def2TZVP)[S5] and compared to each other. Both methods revealed an highly increased steric energy for the anthracene by Höger et al., the first method revealed an increase of 44 kcal/mol (steric energy) compared to **6**, while the second method revealed an increase of 54 kcal/mol (total energy).

2.6 Crystallographic data

Crystals of **5**: Plank-shaped, colorless single crystals were obtained by slow evaporation of a concentrated CH_2Cl_2 solution.

Colourless crystal (plank), dimensions 0.110 x 0.044 x 0.015 mm^3, crystal system triclinic, space group P $\bar{1}$, Z=1, *a*=10.2901(5) Å, *b*=12.8491(7) Å, *c*=13.6417(7) Å, α=78.112(4) °, β=83.437(4) °, γ=82.864(4) °, V=1744.01(16) Å^3, ρ=1.256 g/cm^3, T=200(2) K, θ_{max}= 69.182 °, 17025 reflections measured, 6260 unique (R_{int}=0.0444), 3855 observed (I > 2σ(I)), μ=3.28 mm^{-1}, T_{min}=0.76, T_{max}=10.42, 431 parameters refined, hydrogen atoms were treated using appropriate riding models, goodness of fit 1.04 for observed reflections, final residual values R1(F)=0.058, wR(F^2)=0.151 for observed reflections, residual electron density -0.51 to 0.43 eÅ^{-3}.

Crystals of **6**: Brick-shaped, pale yellow single crystals were obtained by slow evaporation of a concentrated THF solution.

Pale yellow crystal (brick), dimensions 0.057 x 0.027 x 0.020 mm^3, crystal system orthorhombic, space group Pna2$_1$, Z=4, *a*=19.7269(11) Å, *b*=25.1834(13) Å, *c*=14.2934(8) Å, α=90 °, β=90 °, γ=90 °, V=7100.8(7) Å^3, ρ=1.145 g/cm^3, T=200(2) K, θ_{max}= 47.235 °, 15722 reflections measured, 5980 unique (R_{int}=0.0444), 2458 observed (I > 2σ(I)), μ=0.51 mm^{-1}, T_{min}=0.70, T_{max}=1.40, 850 parameters refined, hydrogen atoms were treated using appropriate riding models, goodness of fit 0.82 for observed reflections, final residual values R1(F)=0.055, wR(F^2)=0.058 for observed reflections, residual electron density -0.16 to 0.15 eÅ^{-3}.

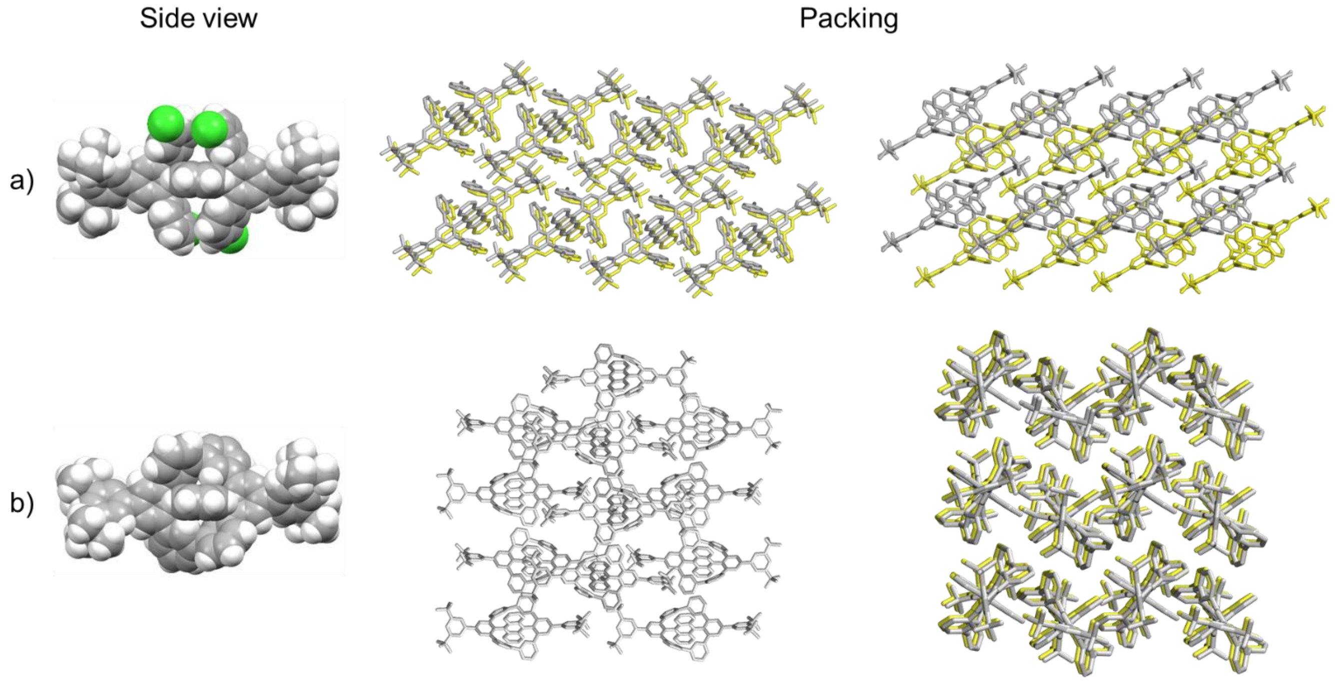

Figure S51: Solid state structures as space-filling model (side view) and packing of **5** (a) and **6** (b). The front layer is shown in grey while the second layer is colored yellow.

Table S2: Crystal data and structure refinement for **5** (CCDC: 2102575).

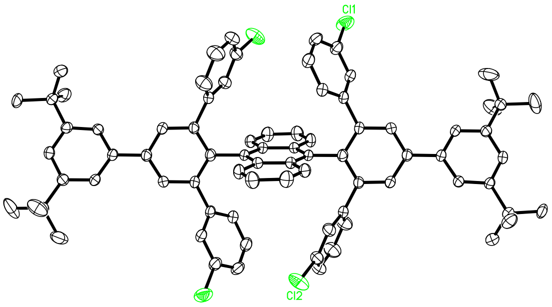

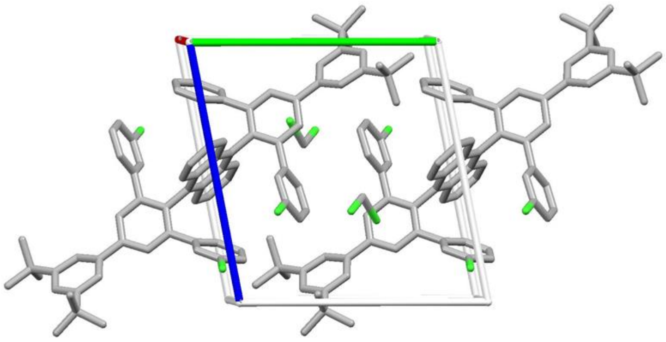

Empirical formula	$C_{80}H_{74}Cl_8$	
Formula weight	1318.99	
Temperature	200(2) K	
Wavelength	1.54178 Å	
Crystal system	triclinic	
Space group	$P\bar{1}$	
Z	1	
Unit cell dimensions	a = 10.2901(5) Å	α =78.112(4) deg.
	b = 12.8491(7) Å	β =83.437(4) deg.
	c = 13.6417(7) Å	γ =82.864(4) deg.
Volume	1744.01(16) $Å^3$	
Density (calculated)	1.26 g/cm^3	
Absorption coefficient	3.28 mm^{-1}	
Crystal shape	plank	
Crystal size	0.110 x 0.044 x 0.015 mm^3	
Crystal colour	colourless	
Theta range for data collection	3.3 to 69.2 deg.	
Index ranges	$-11 \le h \le 12$, $-15 \le k \le 15$, $-16 \le l \le 10$	
Reflections collected	17025	
Independent reflections	6260 (R(int) = 0.0444)	
Observed reflections	3855 ($I > 2\sigma(I)$)	
Absorption correction	Semi-empirical from equivalents	
Max. and min. transmission	10.42 and 0.76	
Refinement method	Full-matrix least-squares on F^2	
Data/restraints/parameters	6260 / 21 / 431	
Goodness-of-fit on F^2	1.04	
Final R indices (I>2sigma(I))	R1 = 0.058, wR2 = 0.151	
Largest diff. peak and hole	0.43 and -0.51 $eÅ^{-3}$	

Table S3: Crystal data and structure refinement for **6** (CCDC: 2102576).

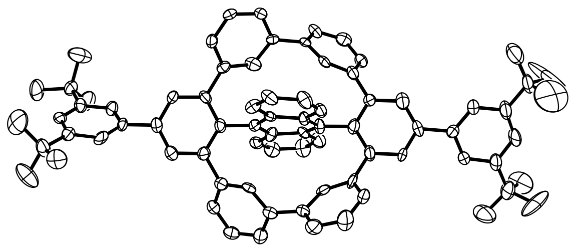

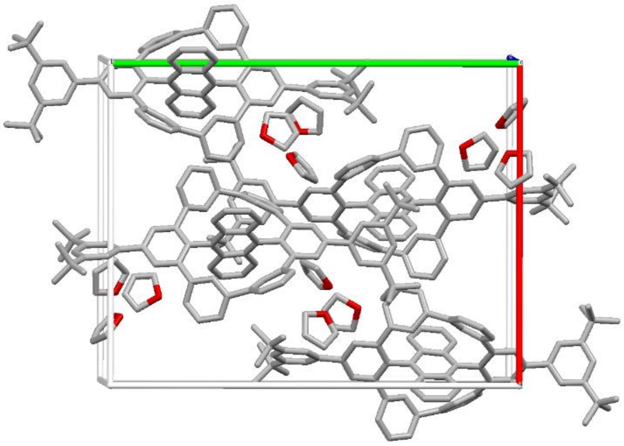

Empirical formula	$C_{90}H_{94}O_3$	
Formula weight	1223.65	
Temperature	200(2) K	
Wavelength	1.54178 Å	
Crystal system	orthorhombic	
Space group	$Pna2_1$	
Z	4	
Unit cell dimensions	a = 19.7269(11) Å	α =90 deg.
	b = 25.1834(13) Å	β =90 deg.
	c = 14.2934(8) Å	γ =90 deg.
Volume	7100.8(7) $Å^3$	
Density (calculated)	1.14 g/cm^3	
Absorption coefficient	0.51 mm^{-1}	
Crystal shape	brick	
Crystal size	0.057 x 0.027 x 0.020 mm^3	
Crystal colour	pale yellow	
Theta range for data collection	2.8 to 47.2 deg.	
Index ranges	$-18\leq h\leq 17$, $-20\leq k\leq 23$, $-13\leq l\leq 13$	
Reflections collected	15722	
Independent reflections	5980 (R(int) = 0.1620)	
Observed reflections	2458 ($I > 2\sigma(I)$)	
Absorption correction	Semi-empirical from equivalents	
Max. and min. transmission	1.40 and 0.70	
Refinement method	Full-matrix least-squares on F^2	
Data/restraints/parameters	5980 / 1255 / 850	
Goodness-of-fit on F^2	0.82	
Final R indices (I>2sigma(I))	R1 = 0.055, wR2 = 0.058	
Absolute structure parameter	-0.1(9)	
Largest diff. peak and hole	0.15 and -0.16 $eÅ^{-3}$	

References

[S1] G. R. Fulmer, A. J. M. Miller, N. H. Sherden, H. E. Gottlieb, A. Nudelman, B. M. Stoltz, J. E. Bercaw, K. I. Goldberg, *Organometallics* **2010**, *29*, 2176-2179.
[S2] G. Sheldrick, *Acta Cryst. A* **2015**, *71*, 3-8.
[S3] G. Sheldrick, *Acta Cryst. C* **2015**, *71*, 3-8.
[S4] W. H. Melhuish, *J. Phys. Chem.* **1961**, *65*, 229-235.
[S5] M. J. Frisch, G. W. Trucks, H. B. Schlegel, G. E. Scuseria, M. A. Robb, J. R. Cheeseman, G. Scalmani, V. Barone, G. A. Petersson, H. Nakatsuji, X. Li, M. Caricato, A. V. Marenich, J. Bloino, B. G. Janesko, R. Gomperts, B. Mennucci, H. P. Hratchian, J. V. Ortiz, A. F. Izmaylov, J. L. Sonnenberg, Williams, F. Ding, F. Lipparini, F. Egidi, J. Goings, B. Peng, A. Petrone, T. Henderson, D. Ranasinghe, V. G. Zakrzewski, J. Gao, N. Rega, G. Zheng, W. Liang, M. Hada, M. Ehara, K. Toyota, R. Fukuda, J. Hasegawa, M. Ishida, T. Nakajima, Y. Honda, O. Kitao, H. Nakai, T. Vreven, K. Throssell, J. A. Montgomery Jr., J. E. Peralta, F. Ogliaro, M. J. Bearpark, J. J. Heyd, E. N. Brothers, K. N. Kudin, V. N. Staroverov, T. A. Keith, R. Kobayashi, J. Normand, K. Raghavachari, A. P. Rendell, J. C. Burant, S. S. Iyengar, J. Tomasi, M. Cossi, J. M. Millam, M. Klene, C. Adamo, R. Cammi, J. W. Ochterski, R. L. Martin, K. Morokuma, O. Farkas, J. B. Foresman, D. J. Fox, Wallingford, CT, **2016**.
[S6] Y. Fujiwara, R. Ozawa, D. Onuma, K. Suzuki, K. Yoza, K. Kobayashi, *J. Org. Chem.* **2013**, *78*, 2206-2212.
[S7] a) J. Shi, C. W. Tang, C. H. Chen*, US5935721A*, United States, **1999**; b) H. Kuma, Y. Jinde (IDEMITSU KOSAN Co., Ltd.)*, US20180208836A1*, United States, **2018**; c) H. Ikeda, M. Ido, M. Funahashi (IDEMITSU KOSAN Co., Ltd.)*, US20060043858A1*, United States, **2006**.
[S8] D. Ryu, E. Park, D.-S. Kim, S. Yan, J. Y. Lee, B.-Y. Chang, K. H. Ahn, *J. Am. Chem. Soc.* **2008**, *130*, 2394-2395.
[S9] B. Dumslaff, M. Wagner, D. Schollmeyer, A. Narita, K. Müllen, *Chem. Eur. J.* **2018**, *24*, 11908-11910.
